# Control of transcription elongation by GreA determines rate of gene expression in *Streptococcus pneumoniae*

**DOI:** 10.1093/nar/gku790

**Published:** 2014-09-04

**Authors:** Yulia Yuzenkova, Pamela Gamba, Martijn Herber, Laetitia Attaiech, Sulman Shafeeq, Oscar P. Kuipers, Stefan Klumpp, Nikolay Zenkin, Jan-Willem Veening

**Affiliations:** 1 Centre for Bacterial Cell Biology, Institute for Cell and Molecular Biosciences, Newcastle University, Richardson Road, Newcastle upon Tyne, NE2 4AX, UK; 2 Molecular Genetics Group, Groningen Biomolecular Sciences and Biotechnology Institute, Centre for Synthetic Biology, University of Groningen, Nijenborgh 7, 9747 AG, Groningen, The Netherlands; 3 Max Planck Institute of Colloids and Interfaces, Science Park Golm, 14424 Potsdam, Germany

## Abstract

Transcription by RNA polymerase may be interrupted by pauses caused by backtracking or misincorporation that can be resolved by the conserved bacterial Gre-factors. However, the consequences of such pausing in the living cell remain obscure. Here, we developed molecular biology and transcriptome sequencing tools in the human pathogen *Streptococcus pneumoniae* and provide evidence that transcription elongation is rate-limiting on highly expressed genes. Our results suggest that transcription elongation may be a highly regulated step of gene expression in *S. pneumoniae*. Regulation is accomplished via long-living elongation pauses and their resolution by elongation factor GreA. Interestingly, mathematical modeling indicates that long-living pauses cause queuing of RNA polymerases, which results in ‘transcription traffic jams’ on the gene and thus blocks its expression. Together, our results suggest that long-living pauses and RNA polymerase queues caused by them are a major problem on highly expressed genes and are detrimental for cell viability. The major and possibly sole function of GreA in *S. pneumoniae* is to prevent formation of backtracked elongation complexes.

## INTRODUCTION

Transcription, the first step of gene expression, is accomplished by highly conserved multisubunit RNA polymerases (RNAPs). Though initiation is the most heavily regulated step of the transcription cycle, accurate and processive elongation of RNA is essential for cell viability and homeostasis. Elongation processivity can be disrupted by pauses including backtracked pauses when the 3′ end of RNA disengages from the active center and RNAP shifts backwards (1). Backtracking is also caused by misincorporation events (2–5). Backtracked complexes can be resolved by hydrolysis of the phosphodiester bond of RNA that re-establishes the 3′ end of RNA in the active center allowing its further elongation. Based on *in vitro* experiments it was shown that RNA hydrolysis by the RNAP active center might contribute to overall fidelity and processivity (2–5).

Intrinsic cleavage activity of the RNAP active center can be greatly stimulated by the evolutionary conserved transcription factor Gre (some bacteria have two factors, GreA and GreB) (3,6). Gre-factor has a long coiled-coil domain, which can bind in the secondary channel toward the RNAP catalytic center. Two conserved acidic residues on the tip of this domain, D41 and E44 (*Escherichia coli* numbering), are thought to stabilize the second catalytic Mg^2+^ ion in RNAPs active center and possibly coordinate the attacking water molecule (3,6–9). Gre was shown to suppress transcription pauses and arrests (10,11), and enhance transcription fidelity *in vitro* (2,3). A *greA* deletion in *E. coli* also strongly affected the bistable regulation of the *lac* operon, which was explained by reduced transcription fidelity *in vivo* (12). Overexpression of *E. coli* GreA (GreA*_Eco_*) resulted in upregulation of more than 100 genes (∼2.4% of the genome) (13). This regulation was proposed to be accomplished through stimulation of transition from transcription initiation to elongation; i.e. promoter escape. In accordance with this idea, an increase in the amount of abortive transcripts was demonstrated in the absence of GreA*_Eco_* (14) and stimulation of promoter escape was suggested to be the major role of Gre in cells in general (13).

Most information on the *in vivo* function of Gre comes from studies using *E. coli*. Interpretation of the effects of Gre mutants in *E. coli* are hampered by the presence of two Gre-factors (GreA*_Eco_* and GreB*_Eco_*) and a number of factors that potentially can also bind and modulate RNAP through the secondary channel (e.g. DksA, Rnk and TraR) (15–18). The functions of these proteins are at least partially redundant. For example, the growth deficiency of a *dksA* mutant was complemented by multicopy *greA* and *greB* (18). Limited functional studies have been performed with bacteria besides *E. coli*; a chromatin immunoprecipitation study showed that *Bacillus subtilis* GreA is uniformly distributed over actively transcribed regions and that its inactivation resulted in the accumulation of RNAP at many promoter or promoter–proximal regions. However, no change in gene expression or phenotype was observed (19). Thus, it is clear that a full functional analysis, both *in vitro* and *in vivo*, of a nonredundant Gre-factor is missing to identify the main *in vivo* role of bacterial Gre -factors.

**Figure 1.**
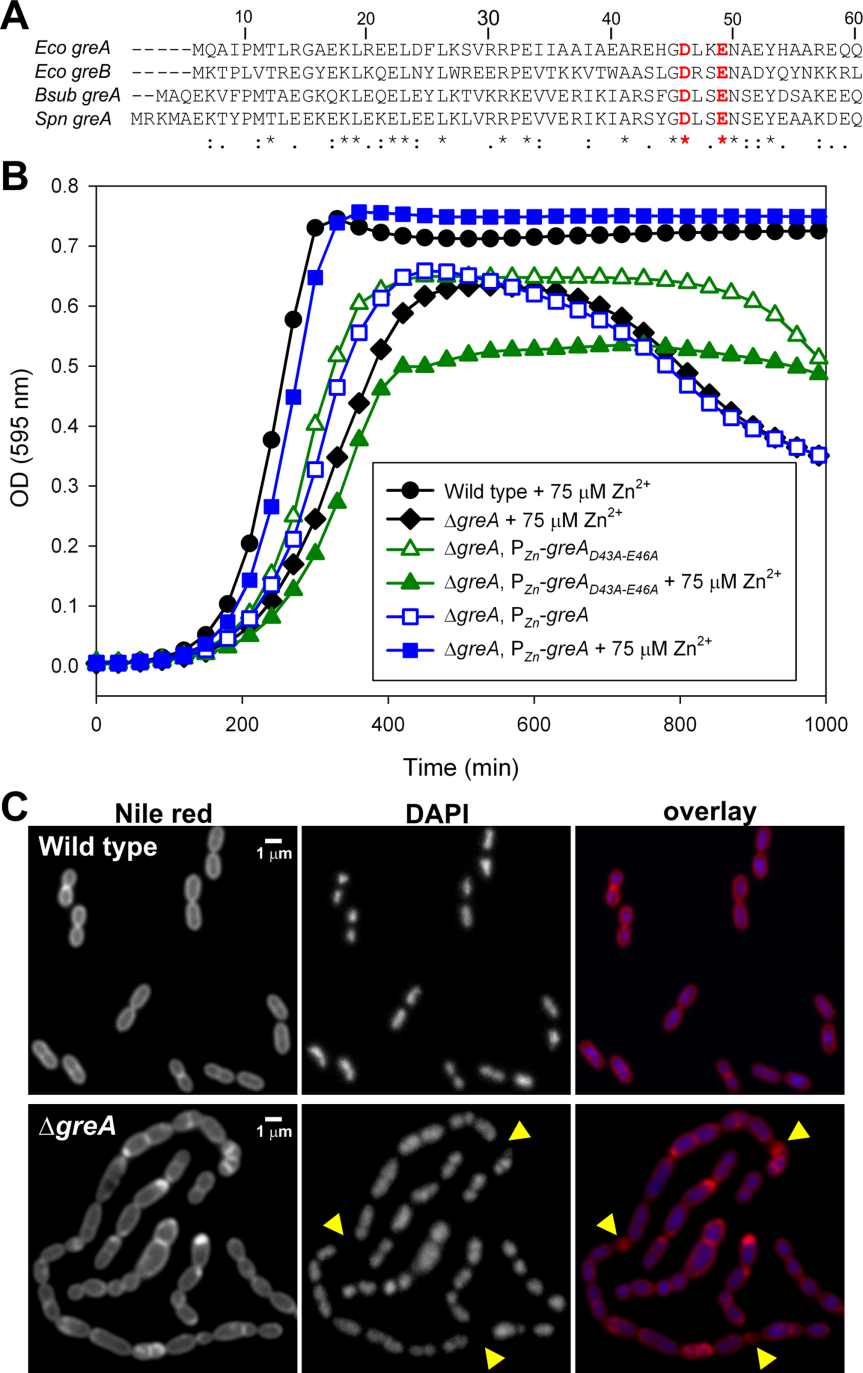
GreA*_Spn_* is crucial for normal growth and cell physiology. (**A**) Sequence alignment of the N-terminal peptides in GreA. The conserved acidic residues crucial for stimulating RNAP hydrolysis are highlighted in red. Abbreviations of species: *Eco*, *Escherichia coli*; *Bsub*, *Bacillus subtilis*; *Spn*, *Streptococcus pneumoniae.* (**B**) Growth curves of wild type and *greA_Spn_* mutant strains grown in C + Y medium. For clarity only every third data point is plotted. Curves are averages of at least three replicates. Note that the optical density is plotted on a linear scale instead of a log scale to better highlight the differences in cell densities at later time-points when the *greA_Spn_* mutant lyses. (**C**) Microscopy analysis of wild type and the *greA_Spn_* mutant. Yellow arrowheads highlight anucleate cells.

The genome of the Gram-positive human pathogen *Streptococcus pneumoniae* only contains a single Gre-factor, GreA*_Spn_*, while no homologs of Gre or other known transcription factors that bind to RNAP's secondary channel have been identified (20,21). GreA*_Spn_* contains the two conserved acidic residues present in all Gre-factors that are required for their activity (Figure 1A). A reduced genome of just 2 million base pairs together with its genetic amenability (22), makes *S. pneumoniae* an excellent model to study the physiological role of bacterial Gre-factors. Using transcriptome sequencing (RNA-Seq), newly developed bioinformatics tools, *in vitro* and *in vivo* analysis and mathematical modeling we show that the major *in vivo* function of GreA*_Spn_* is to prevent long-living pauses which cause queuing or traffic jams of RNAPs and dramatically hamper gene expression.

## MATERIALS AND METHODS

### Strains, plasmids and growth conditions

Bacterial strains and plasmids used in this study are listed in Supplementary Table S5. The oligonucleotides used in this study are listed in Supplementary Table S6. *Streptococcus pneumoniae* strains were grown as standing cultures in complex C + Y medium (23) at 37°C. Detailed growth conditions are described in the supplemental information as well as the construction of the plasmids and strains used.

### Protein purification

*Streptococcus pneumoniae* RNAP was purified by Polymin P, heparin and MonoQ chromatographies as described in (24). *Streptococcus pneumoniae* σ^A^ and GreA (wild-type and mutant) factors were cloned (primers rpoD start nde/rpoD sp xho r and greA start/greA end; Supplementary Table S6) and expressed using the pET expression system and purified as described in (6,25).

### *In vitro* transcription assays

For transcription from promoters, 0.1 pmole of polymerase chain reaction (PCR) fragment carrying promoter (obtained with oligonucleotides from Supplementary Table S6) was mixed with 0.3 pmole of RNAP*_Spn_* and 0.9 pmoles of σ^A^ with or without 0.1 pmole of GreA*_Spn_* in transcription buffer (TB; 33 mM Tris-Ac pH 7.9, 100 mM KGlu, 10 mM MgAc, 0.5 mM DTT, 0.1 mg/ml bovine serum albumin). All reactions were performed at 37°C. After 10 min of open complex formation, reactions were started by addition of 0.5 mM ATP, GTP and CTP and 0.15 mM [α-^32^P]-UTP (7.5 Ci/mmol) final for run-off transcription. For abortive initiation, assay reactions were started with 50 μM dinucleotide primer and 20 μM [α-^32^P]-NTP (125 Ci/mmol). For the promoter of SP_0267, UpA and UTP were used, for both *ccpA* and *purC*, ApA and GTP were used. Reactions were incubated for 10 min and stopped by addition of formamide-containing buffer. Products were resolved by denaturing (8 M urea) PAGE and revealed by Phosphorimaging (GE Healthcare).

Elongation complexes were assembled in TB lacking Mg^2+^ with 13 nt-long 5′ end radiolabeled RNA as described in (5), except that complexes were immobilized on streptavidin agarose beads (Fluka) through biotin of the 5′ end of the DNA template strand (3). To form misincorporated complex (mEC14), 10 mM ATP and 20 mM MgCl_2_ were added for 30 s. After that, 1 mM NTPs were added in the presence or absence of 10 nM GreA*_Spn_* for times indicated in the figure. Reactions were stopped and products analyzed as above.

Permanganate footprinting of open complexes was performed on promoters ^32^P-labeled on either template or nontemplate strand, by addition of 5 mM KMnO_4_ for 30 s, in the presence of 0.5 mM GTP (initiating nucleotide). Reactions were terminated by addition of β-mercaptoethanol (330 mM), followed by phenol-extraction, ethanol-precipitation and 10% piperidine treatment.

### *In vivo* measurement of transcription rate

Velocity of transcription *in vivo* was measured essentially as described in (26). Briefly, cells were grown in C + Y medium to OD_600_∼0.25, transcription of P*_ssbB_-luc-gfp* was induced by adding CSP to a final concentration of 100 ng/ml. Samples were withdrawn with 10-s intervals and transcription was stopped by adding double volume of RNA Protect reagent (Qiagen). Total RNA was extracted by the hot phenol method and 5 μg was used for each dot on northern dot-blot. Early and late RNA probes were obtained by T7 RNA polymerase on PCR templates made with oligonucleotides luc early start/luc early end t7 (early probe) and gfp late start/gfp late end t7 (late probe). Dot-blotting was performed and analyzed as described in (26).

### Fluorescence microscopy

Cells were grown at 37°C in plastic 5 ml capped tubes, basically as described previously (27). Microscopy pictures were taken with a Deltavision (Applied Precision) IX71Microscope (Olympus), using a CoolSNAP HQ2 camera (Princeton Instruments) and a 300 W Xenon light source through a 100x oil immersion objective (phase contrast). For more details, see the supplementary information.

### RNA-Seq

Total RNA was isolated from mid exponentially growing cells and cDNA sequence libraries were prepared and sequenced as described in detail in the supplemental information. Raw sequence data are deposited and available on Sysmo-Seek (https://seek.sysmo-db.org Project Noisy Strep).

### Calculating transcription mistakes using RNA-Seq data

Illumina raw sequence error rates are quite high, which might be caused by natural errors, errors introduced during cDNA library construction and Illumina-specific errors (28–30). Therefore, raw RNA-Seq data were first filtered to exclude all reads containing stretches of five or more similar nucleotides in a row (e.g. TTTTT). Furthermore, only high quality reads were selected by trimming all reads indiscriminately to 95 bp (removing the first and last 20 bases) and using the fastx toolkit to reject reads on quality (average phred score 25 and no phred scores below 15). Then the reads for both wild type and *greA* mutant were aligned using the Bowtie readmapper (31). The resulting mapped reads were compared with the reference genome of *S. pneumoniae* D39 (NCBI annotation ID NC_008533.1), read by read, position by position using a custom C++ program. All bases in the reads different from the reference were tallied and an average per read was calculated. These averages of mismatch rates were a mix between transcription errors and sequencing errors. It should be valid to compare them between wild type and mutant because sequencing errors should on average be the same given the same sequencing procedure used for both wild type and mutant samples. This approach was further validated by the fact that error rates were found to be significantly higher when cDNA libraries were prepared from the same RNA sample using lower fidelity reverse-transcriptase enzymes.

### *In vivo* fidelity assay

Nonsense suppression was measured by determining β-galactosidase activity in cultures of pneumococcal strains carrying a multicopy plasmid containing a *lacZ* reporter gene with a premature stop codon early in the coding sequence. Strains were grown at 37°C in C + Y medium supplemented with 0.15 μg/ml erythromycin. At appropriate optical density (O.D. 550 = 0.3), cells were harvested, concentrated 10 times in Z-buffer (60 mM Na_2_HPO_4_, 40 mM NaH_2_PO_4_, 10 mM KCL, 1 mM MgSO_4_), flash frozen in liquid nitrogen and stored at −20°C. To determine β-galactosidase activity, samples were thawed at room temperature and mixed with 30 ug/ml hexadecyl trimethylammonium bromide. After 5 min of incubation at 30°C, o-nitrophenyl-β-d-galactopyranoside was added to a final concentration of 364 μg/ml. Incubation was continued at 30°C and reactions were stopped by addition of 0.23 volumes of 1 M Na_2_CO_3_. Absorbance was measured at 420 nm. Miller units of β-galactosidase activity were calculated according to the formula: (522 × A420 nm)/(time(min) × volume (ml) × O.D. 550). It should be noted that similar results were obtained by using the protein levels, as determined by a Bradford assay, instead of the O.D. of the samples at time of collection.

### Simulations of the stochastic transcription model

The model of Klumpp and Hwa (32) was adjusted in the following way (also see Supporting text): Elongation complexes are described as stochastic steppers on a one-dimensional lattice. They enter the system at the promoter with the initiation attempt rate α if the promoter is free, step forward with the elongation rate ϵ and leave the system when reaching the termination site. All steps are rejected if the target site is occupied by another elongation complex. At specific (randomly selected) sites, elongation complexes may undergo transitions to a stalled state with rate *f*, from which they are rescued with rate 1/τ, where τ is the duration of the stall event. The effect of GreA*_Spn_* is described as a strong reduction of τ (or, in alternative scenarios, as a reduction of *f* or an increase of ϵ). The model was simulated with the kinetic Monte Carlo approach described in (32), with a basic time step of 0.01 s. Elongation measurements were simulated by starting with an empty lattice and averaged over 1000 simulation runs. To study the dependence on expression level (modulated by varying α) and the gene length, the simulations were allowed to reach the steady state and transcription rates were obtained as time averages over 4.5 × 10^6^ Monte Carlo steps.

## RESULTS

### GreA*_Spn_* is crucial for cellular growth and cell morphology

To characterize the function of GreA*_Spn_ in vivo* we replaced *greA_Spn_* with a chloramphenicol resistance cassette resulting in strain PGs6 (see supplementary methods). Besides reduced growth within agar plates (not shown), Δ*greA_Spn_* cells showed a significant increased doubling time in liquid C + Y media (43 ± 3 min for Δ*greA_Spn_* versus 28 ± 1 min for wild type, ± indicates standard deviation). Furthermore, cultures did not reach the same cell density as wild-type cells, and OD_600_ started to drop after prolonged incubation (Figure 1B). Microscopy analyses (not shown) revealed that the drop in OD_600_ was caused by cell lysis rather than by cell clumping. Note that these growth curves were started by diluting exponentially growing cells, and not directly from frozen stocks and thus do not reflect a decreased ability to survive freezing.

To exclude the possibility that the observed growth defects were due to a polar effect of the chloramphenicol resistance cassette, we introduced a copy of *greA_Spn_* at the ectopic, nonessential, *bgaA* locus, under the control of a Zn^2+^ inducible promoter (P*_Zn_*), resulting in strain PGs48 (Δ*greA_Spn_*, *bgaA*::P*_Zn_*-*greA_Spn_*). In the presence of 75 μM of Zn^2+^, normal cell growth was restored (Figure 1B). Wild-type cells grew identically in the absence or presence of added Zn^2+^ (data not shown). To test whether the catalytic activity of GreA*_Spn_* is required for normal growth, we cloned the catalytically inactive *greA_Spn-_*_D43A/E46A_ mutant allele under the control of P*_Zn_* and integrated this construct at the *bgaA* locus in an otherwise Δ*greA_Spn_* background (strain PGs67: Δ*greA_Spn_*, *bgaA*::P*_Zn_*-*greA_Spn-_*_D43A/E46A_). Strikingly, induction of GreA*_Spn-_*_D43A/E46A_ with 75 μM of Zn^2+^ resulted in even stronger growth defects than without zinc (Figure 1B), suggesting that the presence of inactive GreA at RNAPs catalytic site perturbs RNA polymerase functions. This observation is consistent with our earlier results, which showed that catalytically deficient GreA further ‘switches off’ intrinsic hydrolytic activity of RNAP by sequestering the Trigger Loop of the active center (3). However, although we used the minimal concentration of Zn^2+^ sufficient for full complementation in PGs48, we cannot formally exclude the possibility that zinc-induced GreA*_Spn-_*_D43A/E46A_ is present at higher than wild-type GreA levels. Note that for reasons currently unknown, cell lysis after prolonged incubation (>600 min) was less pronounced in strain PGs67 (Δ*greA_Spn_*, *bgaA*::P*_Zn_*-*greA_Spn-_*_D43A/E46A_) compared to the *greA* mutant (PGs6) (Figure 1B).

To examine the effects of *greA_Spn_* deletion on cell morphology, strain PGs6 (Δ*greA_Spn_*) and the wild type parental strain (D39) were grown in liquid C + Y medium at 37°C and cells were harvested for microscopy at mid-exponential growth. DNA was stained with 4',6-diamidino-2-phenylindole (DAPI) and membranes were stained with the lipophilic Nile red dye. As shown in Figure 1C, Δ*greA_Spn_* cells exhibited a pleiotropic array of cell morphologies including chains of cells, small cells and large cells. In line with a defect in transcription, DAPI staining showed the occasional presence of anucleate cells in the Δ*greA_Spn_* mutant (∼2.8%, >1000 cells counted, (33)), whereas this was never the case for the wild type (Figure 1C). Complementation of Δ*greA_Spn_* with 75 μM of Zn^2+^ in the PGs48 strain resulted in normal cell morphology (Supplementary Figure S1). Together, these data demonstrate that the activity of GreA*_Spn_* is crucial for normal growth and cell physiology in *S. pneumoniae*.

### Absence of GreA*_Spn_* slightly increases *in vivo* error rate in gene decoding

**Figure 2.**
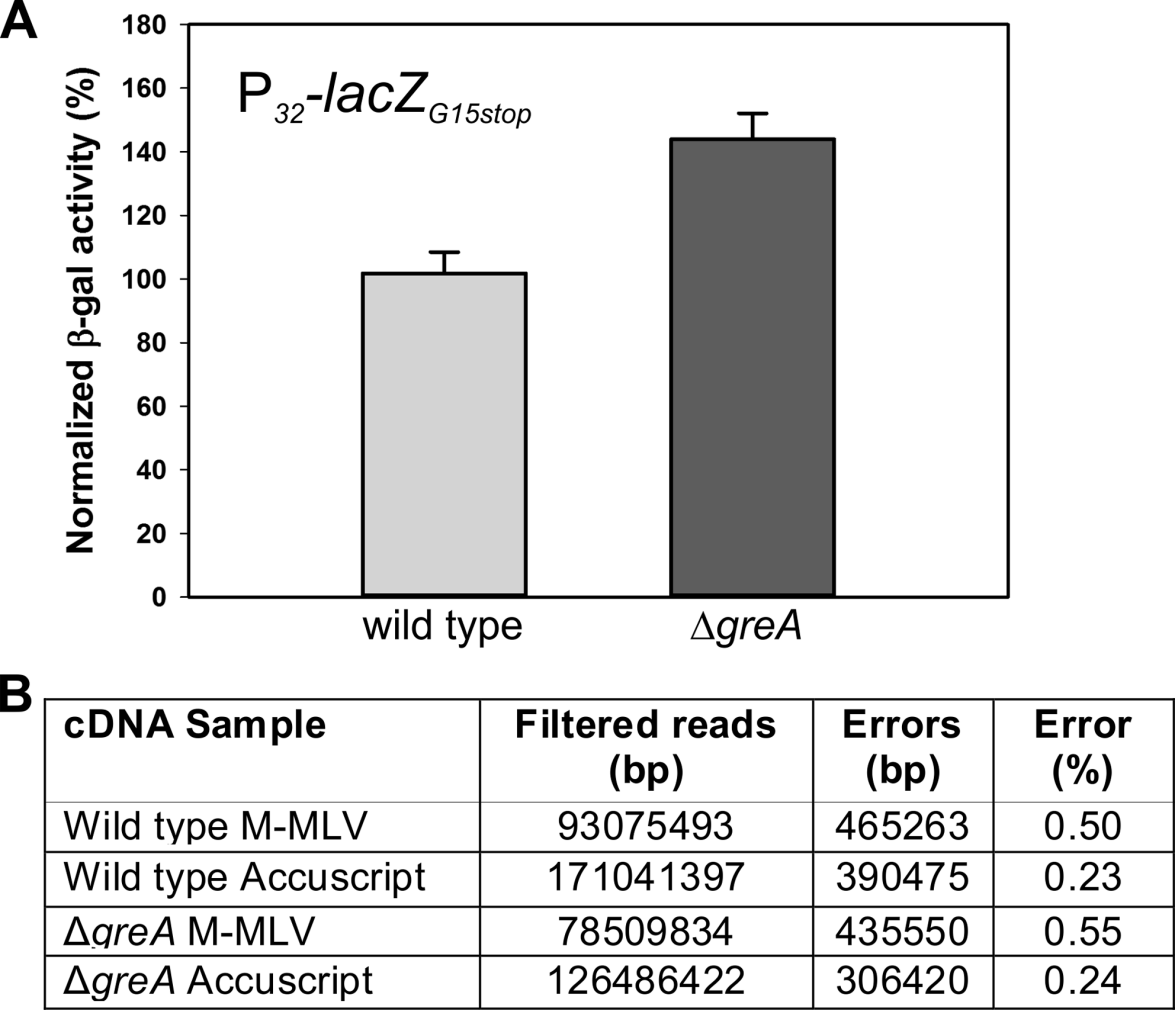
Absence of GreA*_Spn_* slightly increases *in vivo* error rate in gene decoding. (**A**) Normalized β-galactosidase activity levels of a wild type and a Δ*greA* strain carrying a multicopy plasmid with a constitutively expressed *lacZ* reporter gene with a premature stop codon early in the coding sequence. Values shown are averages of three independent replicates (error bars: standard deviation). (**B**) RNA-Seq data were filtered to only include high quality reads. Transcription errors were tallied using a custom Python script by comparing the sequence of the mapped reads to the corresponding positions in the reference genome of *S. pneumoniae* D39 and the percentage of high quality reads that misaligned are shown (see Materials and Methods).

*In vitro* data suggest that Gre-factors play an important role in transcription fidelity (2,3). The above-mentioned results show that cells lacking *greA_Spn_* are significantly perturbed in their physiology. This may be caused by an increased rate of transcriptional errors. To directly examine if transcription fidelity is affected by *greA_Spn_* deletion *in vivo*, we constructed a reporter cassette that contains a constitutively expressed *lacZ* gene containing a stop codon mutation (P*_32_*-*lacZ_G15stop_*). Functional LacZ will thus only be produced if errors in transcription or translation are regularly made. The *lacZ-*fidelity reporter was introduced on a replicative multicopy vector, resulting in plasmid pPGs6. The *bgaA* locus, encoding the only endogenous galactosidase of *S. pneumoniae*, was deleted in both wild type and Δ*greA_Spn_* to reduce background activity in LacZ assays. As shown in Figure 2A, a small, though significant, difference in production of functional LacZ was observed in the absence of GreA*_Spn_* (<145% of wild type; *P* < 0.05, *t*-test, 8 replicates) and fidelity could be restored by GreA*_Spn_* complementation (Supplementary Figure S2).

To further investigate the effects of GreA*_Spn_* deletion on fidelity of transcription, we performed transcriptome sequencing (RNA-Seq). Total RNA was isolated from mid-exponentially growing cultures of wild type (D39) and Δ*greA_Spn_* (PGs6) in C + Y medium at 37°C and sequencing libraries were prepared (see Materials and Methods). To assess whether this methodology would allow us to detect transcription mistakes, we reverse transcribed total RNA with Moloney murine leukemia virus (M-MLV) reverse transcriptase (RT) and with a variant of the same enzyme called AccuScript RT which has approximately 3- to 6-fold increased fidelity according to the supplier (Agilent). The cDNA libraries were subsequently sequenced using Illumina sequencing (see Materials and Methods for details). After rigorous filtering of the reads (see Materials and Methods), we found that the cDNA libraries prepared with AccuScript RT had an overall lower mismatch rate when aligned to the reference genome sequence (GenBank Acc. NC_008533.1) compared to the cDNA libraries prepared with M-MLV (Figure 2B) thus validating this approach. If the Δ*greA_Spn_* mutant generates more mistakes during transcription than the wild type, this should be reflected in an increase of the overall mismatch rate of the RNA-Seq reads. Indeed, we found an overall mismatch rate of 0.24 and 0.55% in the Δ*greA_Spn_* mutant of cDNA libraries prepared with either AccuScript or M-MLV compared to 0.23 and 0.50% of errors in the wild type demonstrating slightly more transcription errors in the absence of GreA*_Spn_* (Figure 2B). The observed mismatch rate is much higher than previously determined transcription error rates (∼10^−5^ per nucleotide; (34)) which are generally based on assays similar as the aforementioned *lacZ* assay and are thus difficult to extrapolate over the entire transcriptome. However, the error rate found here is exaggerated by mistakes introduced during reverse transcription, PCR and the process of Illumina sequencing itself (also see Supplementary Material). Because of the small differences observed by the RNA-seq analysis we cannot make any quantitative conclusions regarding the extent of reduced fidelity of the *greA* mutant. However, Stevens *et al.* showed that a 2-fold increase in decoding errors (which is much larger than observed here) causes only mild negative effects on pneumococcal cell growth (35). Taken together, we can infer that reduced transcription fidelity cannot be the major source of the observed pleiotropic effects on cell physiology of Δ*greA_Spn_* cells. Note, however, that misincorporation events lead to paused complexes formation, which may have more detrimental effects than the RNA sequence alterations (see below).

### Highly expressed genes are more sensitive to lack of GreA*_Spn_*

Analysis of the RNA-Seq data revealed that more than 25% of the genome was more than 2-fold differentially expressed in the Δ*greA_Spn_* mutant (Supplementary Table S1). Roughly half of these genes were more than 2-fold upregulated and about half were more than 2-fold downregulated (Supplementary Table S1). Real-time quantitative PCR on RNA isolated from wild type and Δ*greA_Spn_* cells using primers for a selected set of genes verified the RNA-Seq results (Supplementary Table S2). Among the upregulated genes were the genes belonging to the CiaR/H regulon (e.g. *htrA*, *SPD_0775* and *SPD_0913*), which is activated upon envelope stress (36,37); the heat shock induced HrcA regulon (e.g. *clpL* and *dnaK*) and genes involved in DNA-repair (e.g. *dprA*, *ssb* and *SPD_0715*) indicating a potential conflict between replication and transcription in the absence of GreA*_Spn_* (Supplementary Table S3). Also, the *ccpA* gene, encoding for the global catabolite repressor protein was downregulated (Supplementary Table S2). Likely because of *ccpA* downregulation, over one third (38 genes) of its core regulon (38) were subsequently more than 2-fold up- or downregulated (Supplementary Table S3). Perturbed expression of this global regulator alone could already account for more than 7% of all the differentially regulated genes in the Δ*greA_Spn_* mutant (Supplementary Table S3). Other noteworthy downregulated genes are involved in DNA-replication (e.g. *purC*, *ogt* and *dnaX*), cell wall synthesis (e.g. *glmM*, *pbp1A* and *pbp1B*) and protein synthesis (e.g. *rpsT, efTU* and *prfC*). Potential differences in mRNA stability in the Δ*greA_Spn_* mutant are unlikely to affect the expression patterns, as it was shown that, at any growth phase, the impact of synthesis greatly outweighs the impact of degradation on the level of all mRNAs studied (39).

**Figure 3.**
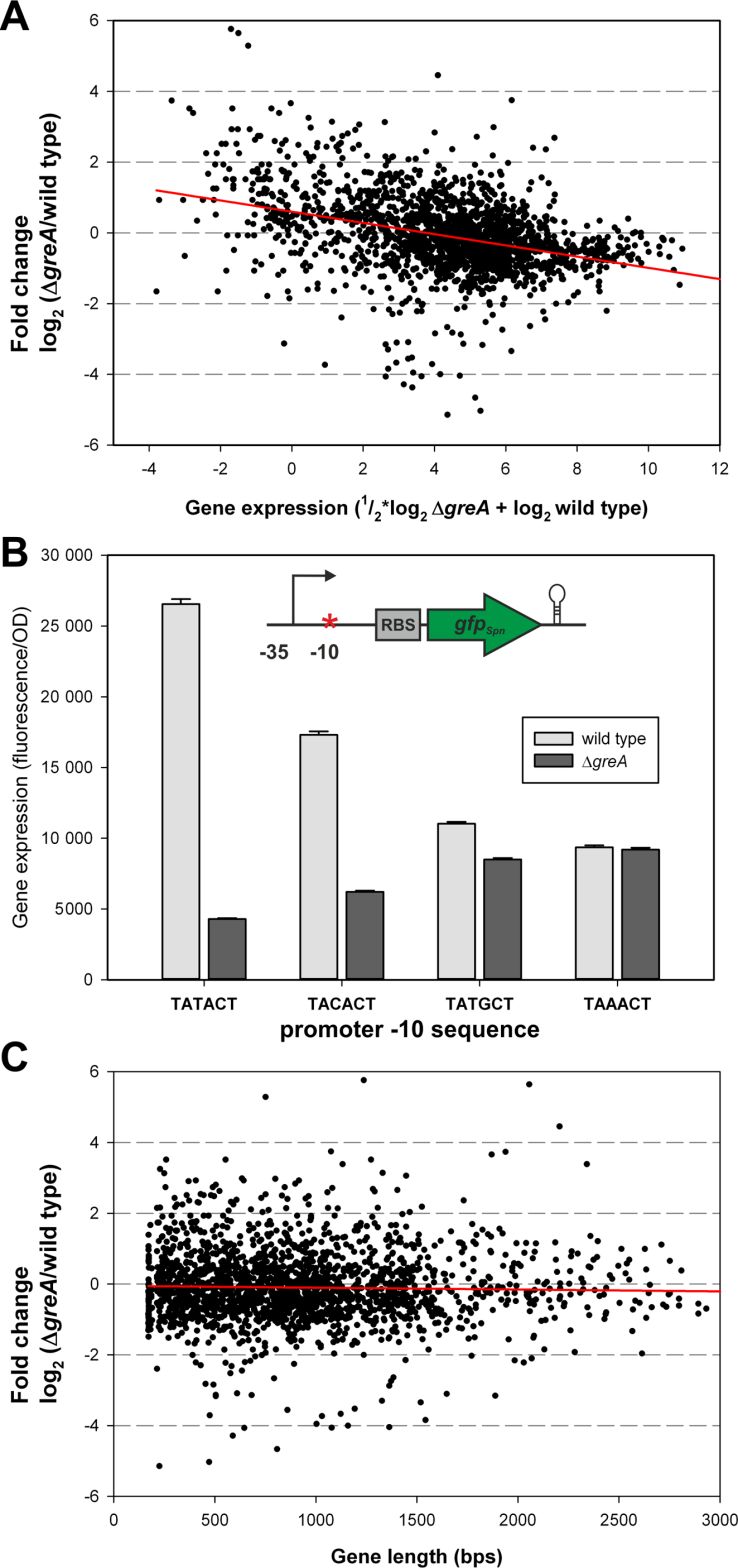
Highly expressed genes are more sensitive to lack of GreA*_Spn_*. (**A**) RNA-Seq data plotted as an MA-plot. Each dot represents the expression value and fold change of a single Open Reading Frame (ORF). Note that some outliers fall outside the plotted area (<1% of all data points). A linear regression line (*R* = 0.36) is shown in red. (**B**) Effects of Δ*greA_Spn_* on the expression of Green Fluorescent Protein (GFP) driven by synthetic constitutive promoters. Expression of GFP is disproportionally reduced when the promoter is stronger in the Δ*greA_Spn_* mutant and the differences are smaller when the promoter is less strong. (**C**) Differential expression in the Δ*greA_Spn_* mutant is not correlated with gene length.

Interestingly, when we plotted the fold change difference between Δ*greA_Spn_* and wild type as a function of gene expression strength (similar to a MA-plot (40)), a clear trend is visible in that highly expressed genes were affected more by the absence of GreA*_Spn_* than lowly expressed genes (Figure 3A, *R* = 0.36). Note however that this trend did not hold for the strongly expressed ribosomal RNAs (rRNAs) (Supplementary Table S4; see Discussion). To validate this observation, we constructed a set of strains carrying synthetic constitutive promoters of different strengths driving Green Fluorescent Protein (GFP) in both the wild type and Δ*greA_Spn_* genetic backgrounds and measured total fluorescence as a proxy for transcription rate. The effect of GreA*_Spn_* deletion on these promoters reaffirmed the observations at the genomic level: strongly expressed genes were more strongly affected (Figure 3B). Interestingly, we observed no correlation between gene length and GreA*_Spn_* dependency (Figure 3C, *R* = 0.03; see below). Together, these results suggest that, in general, gene expression is reduced in the absence of GreA*_Spn_*, resulting in knock-on effects that may lead to gene upregulation, such as in the case of the CcpA regulon. These global transcriptional changes may ultimately be responsible for the pleiotropic phenotypes displayed by Δ*greA_Spn_* mutant cells (Figure 1).

### Transcription initiation and promoter escape are not influenced by GreA*_Spn_*

**Figure 4.**
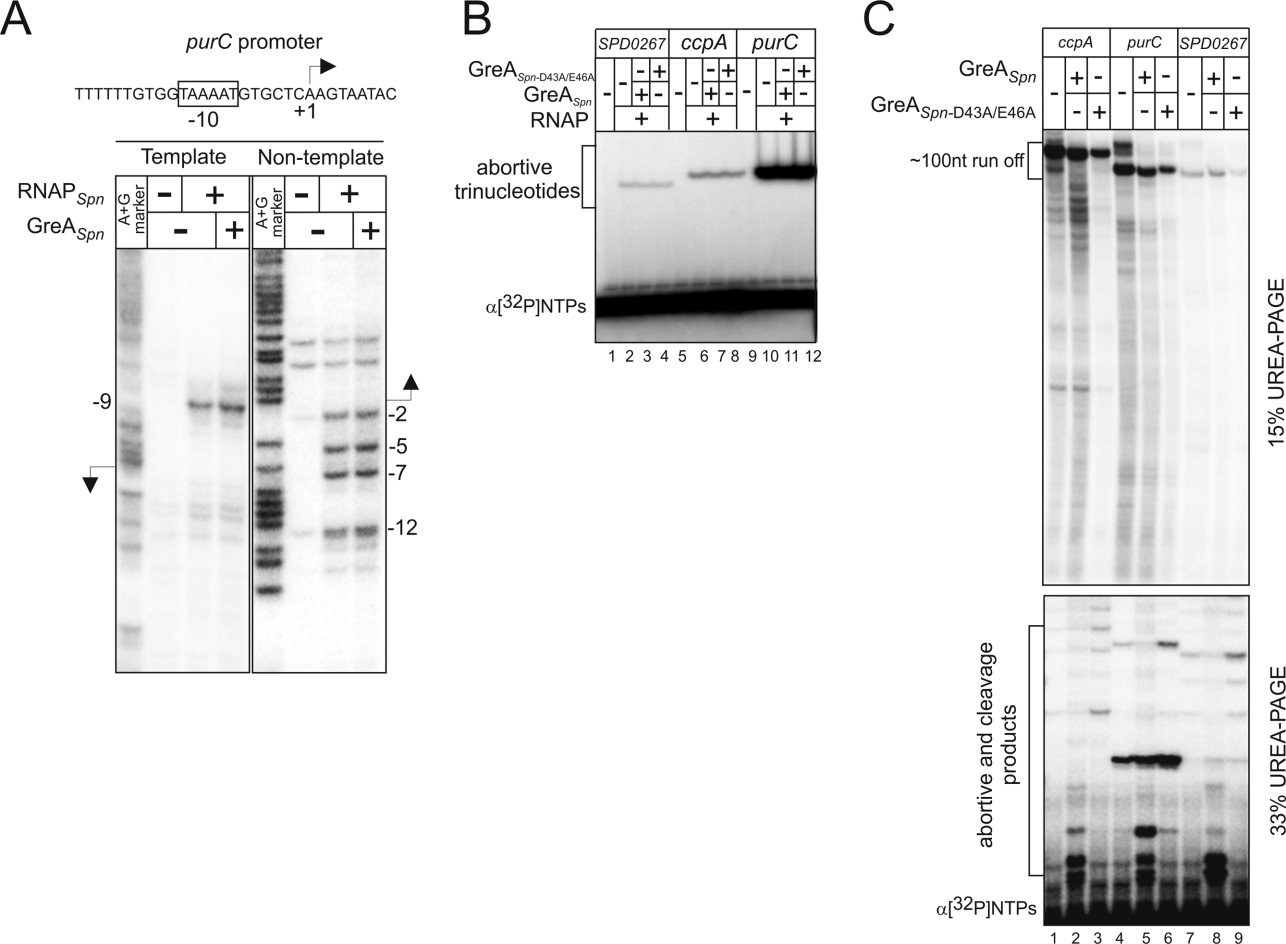
Transcription initiation and promoter escape are not influenced by GreA*_Spn._* (**A**) Open complexes of the *purC* promoter formed by RNAP*_Spn_* in the presence or absence of GreA*_Spn_* were probed with KMnO_4_. A + G reaction was used as a marker. **(B)***In vitro* abortive initiation on *ccpA*, *purC* and *SPD_0267* with or without GreA*_Spn_* or mutant GreA*_Spn-_*_D43A/E46A_. (**C**). Products of *in vitro* transcription on short (resulting in ∼100 nt-long run off) *ccpA*, *purC* and *SPD_0267* with or without GreA*_Spn_* or mutant GreA*_Spn-_*_D43A/E46A_ were separated on 15 and 33% denaturing gels to visualize run off and abortive products, respectively. Short cleavage products at the bottom of the gel in the presence of GreA*_Spn_* originate from cleavage in elongation complexes, as no cleavage is seen in the abortive initiation assay (panel B). Additional low mobility bands in the presence of GreA*_Spn_* are thought to be cleavage products of the longer transcripts, in particular the full length ones that are known to be retained in the elongation complex at the ends of templates. Note that pauses in the presence or absence of GreA*_Spn-_*_D43A/E46A_ are similar and the apparent differences are attributed to the contrast of the image.

We next analyzed the steps of the transcription cycle (initiation, promoter escape or elongation) affected by the absence of GreA*_Spn_*. First, we tested if the absence of GreA*_Spn_* influences open promoter complex formation *in vitro* using purified GreA*_Spn_* and holo RNAP*_Spn_.* We used a PCR fragment carrying the promoter of the *purC* gene, which, according to the RNA-seq and qRT-PCR data, was strongly affected by GreA*_Spn_* deletion (Supplementary Tables S2 and S3). Open complexes were probed with KMnO_4_, which modifies thymine bases only in single stranded regions of DNA. As seen from Figure 4A, the presence of GreA*_Spn_* did not influence open complex formation *in vitro*.

Next, we analyzed abortive initiation with or without GreA*_Spn_* or mutant GreA*_Spn-_*_D43A/E46A_ on several templates (*ccpA*, *purC* and *SPD_0267*) whose gene expression was decreased in the Δ*greA_Spn_* mutant (Supplementary Tables S2–S3). To do so, we monitored extension of a dinucleotide primer with a radiolabeled nucleoside monophosphate. The experiment demonstrated that GreA*_Spn_* does not affect this stage of transcription (Figure 4B). GreA from *E. coli* was proposed to increase the efficiency of promoter escape (13). Therefore, we tested *in vitro* transcription on the same templates. As shown in Figure 4C the pattern of abortive transcripts formed during promoter escape or the ratio of abortive transcripts to the run-off RNA (product of transcription till the end of the linear template) were the same in the presence or absence of GreA*_Spn_*. As expected, a number of cleavage products appeared when GreA*_Spn_* was present in the reaction. We therefore conclude that GreA*_Spn_* has no, or minor effects on transcription initiation and promoter escape.

### GreA*_Spn_* stimulates production of full-length transcripts through suppression of transcription pauses

**Figure 5.**
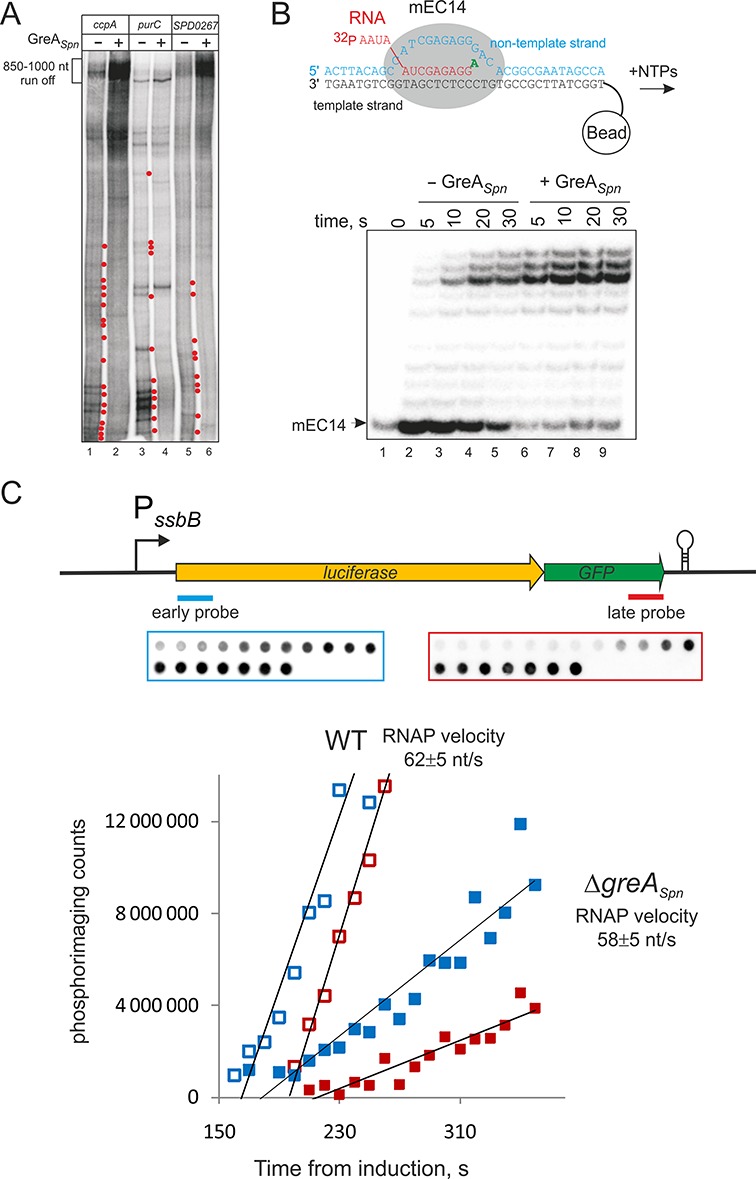
GreA*_Spn_* stimulates elongation. (**A**) Run off *in vitro* transcription with or without GreA*_Spn_* on DNA-templates carrying full-length *ccpA*, *purC* and *SPD_0267* (compare to Figure 4C). Red dots highlight transcription pauses released in the presence of GreA*_Spn_*. (**B**) Elongation complex was assembled from complementary template and nontemplate DNA oligonucleotides and 5′ end labeled RNA. These complexes were forced to misincorporate ATP for 30 s. After that (without washing) NTPs were added in either the presence or absence of GreA*_Spn_*. Note that the fainter mEC14 band at ‘0’ time point is caused by loading. The top bands are a few nucleotides shorter than the expected run off product, likely due to immobilization of the complexes on streptavidin beads through biotin at the 5′. (**C**) To analyze the rate of transcription elongation *in vivo*, total RNA was isolated at various time intervals after induction of the CSP-inducible P*_ssbB_* promoter and northern blotting with probes complementary to the 5′ and 3′ ends of the *luc-gfp* transcript (scheme at the top). Representative dot blots of the early and late probes are shown above the plots. The rate of transcription is calculated as the distance between the probes divided by the time between the emergences of signals of early and late probes. Later emergence of the probes signals in the mutant strain (closed symbols) compared to the wild type (open symbols) could be due to altered timing of induction of the P*_ssbB_* promoter, which involves several steps, which may in their turn be affected by deletion of GreA*_Spn_*. This however does not affect the rate of elongation.

Curiously, the amount of 100 nt run-off RNAs on the templates used in the above experiment was not increased by the presence of GreA*_Spn_* (e.g. compare lanes 1 and 2 in Figure 4C). This result apparently contradicts the RNA-seq data that show increased transcription of these genes in the wild-type strain (in the presence of GreA*_Spn_*). We hypothesized that GreA*_Spn_* action becomes apparent only during transcription of the full-length transcripts, i.e. transcription further downstream of the +100 register. We therefore analyzed transcription *in vitro* on templates carrying full-length *ccpA*, *purC* and *SPD_0267* DNA sequences. In agreement with our hypothesis, in the presence of GreA*_Spn_*, the run-off transcripts on these templates accumulated much more readily than in its absence (Figure 5A). Earlier, we observed that RNAP*_Spn_* has lower processivity on some sequences than bacterial RNAPs from *E. coli* or *Thermus aquaticus* (41). Indeed, specific transcriptional pauses or arrests could be seen in the *in vitro* transcription assays in the absence of GreA*_Spn_* (Figure 5A, red dots).

The *in vivo* analysis suggested that GreA*_Spn_* does not strongly contribute to the sequence correctness of produced RNA, which apparently is achieved by the accuracy of RNAP*_Spn_* active center (42). However, misincorporation events, if not immediately resolved, lead to strong pausing of transcription (2,3,5). We tested if GreA*_Spn_* can suppress misincorporation pausing by stimulating RNAP*_Spn_* to cleave the erroneous RNA. To do so, we used artificial elongation complexes that were assembled from synthetic template and nontemplate DNA oligonucleotides (fully complementary to each other) and 5′ end radiolabeled RNA oligonucleotides (see scheme in Figure 5B). Such complexes were shown to be indistinguishable from the elongation complexes obtained by transcription from a promoter (4,5,43). Assembled RNAP*_Spn_* elongation complexes were forced to misincorporate ATP at the dCMP base in the DNA template and then (without washing the complexes) allowed to elongate in the presence of all NTPs in either the presence or absence of GreA*_Spn_*. Elongation in the absence of GreA*_Spn_* leads to a strong pause in further extension of erroneous transcript (Figure 5B). This pause was absent in the presence of GreA*_Spn_* (Figure 5B) indicating that GreA*_Spn_* can also contribute to processivity of elongation by suppressing the pauses caused by misincorporation as was also shown for *E. coli* GreA and *T. aquaticus* GreA (2,5).

### GreA*_Spn_* does not influence the rate of elongation

So far, our results indicate that GreA*_Spn_* facilitates processivity of transcription elongation by suppressing pausing by RNAP*_Spn_*. However, it is unclear whether this suppression leads to an increased rate of transcription (if GreA*_Spn_* suppresses short-living pauses) or increases the chance of RNAP finishing transcription of a gene; two kinetically distinct scenarios. To distinguish between these scenarios, we examined the influence of GreA*_Spn_* on the velocity of RNAP*_Spn_* elongation *in vivo*. To do so, we compared kinetics of synthesis of 5′- versus 3′-proximal part of an inducible genomic ∼2700 bp long *luc-gfp* reporter transcript in wild type and Δ*greA_Spn_* strains. The method involves isolation of total RNA at various time intervals after induction of the CSP-inducible P*_ssbB_* promoter and northern blotting with probes complementary to the 5′ and 3′ ends of the *luc-gfp* transcript. The time elapsed between the appearances of the two signals after addition of inducer is used to estimate transcription elongation velocity (26,44). As shown in Figure 5C, the elongation kinetics were very similar for both wild type and Δ*greA_Spn_* strains (62 ± 5 versus 58 ± 5 nt/s, respectively), suggesting that GreA*_Spn_* has little effect on the speed of transcribing RNAP*_Spn_*. However, in contrast to the wild type, the slope of the emerging 3′ probe signal is less steep than that of the signal emerging for the 5′ end probe. This cannot be explained by altered Rho-dependent polarity, since *S. pneumoniae* does not have a Rho factor (20,21). Therefore, this result indicates that not all of the RNAPs that started transcription from the promoter were able to reach the terminator in the Δ*greA_Spn_* strain within the time of the experiment. Thus, we conclude that GreA*_Spn_* suppresses the long-living or dead-end pauses, which otherwise preclude RNAP*_Spn_* from finishing transcription.

### Development and characterization of a stochastic model of GreA*_Spn_*-dependent transcription

**Figure 6.**
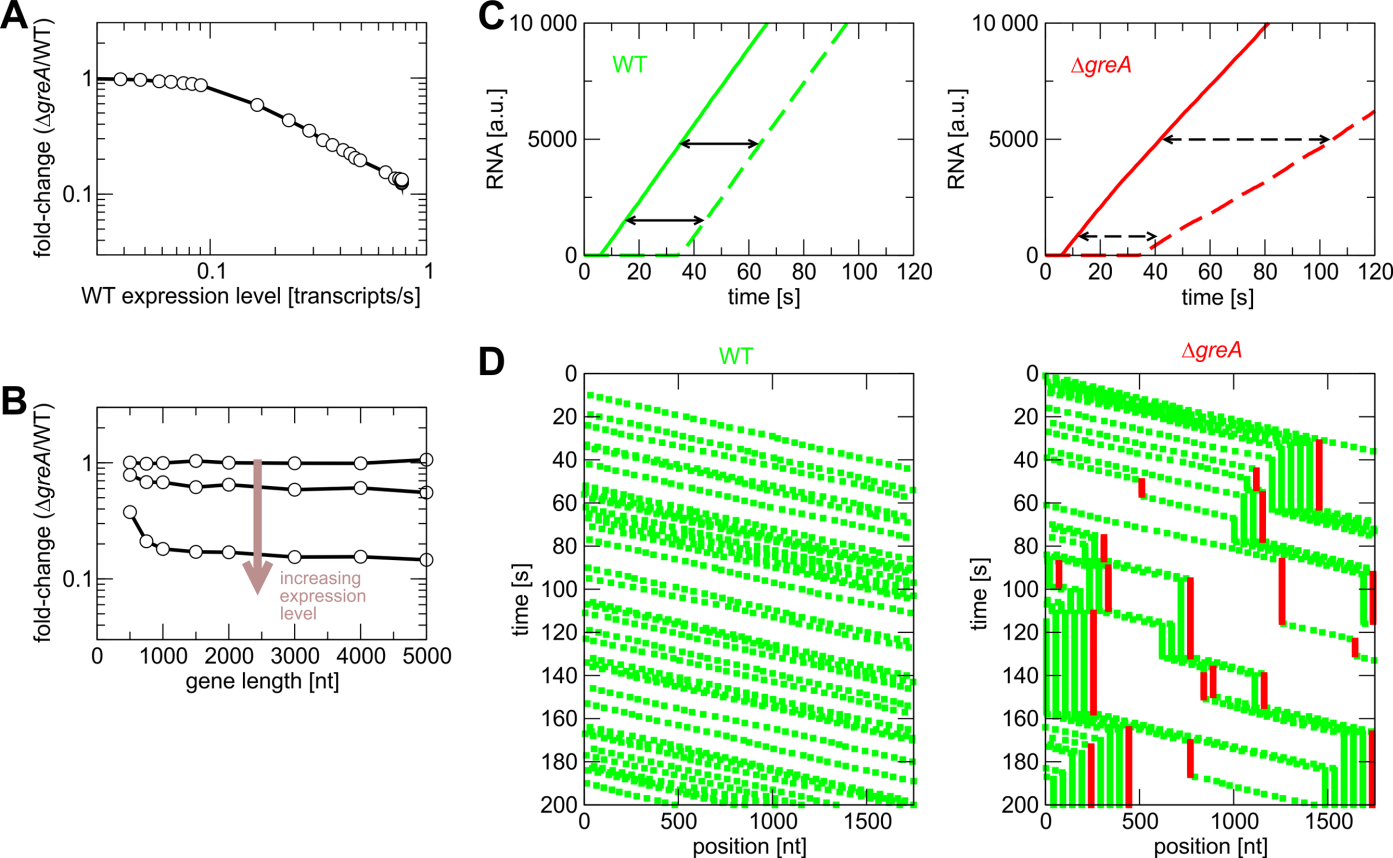
Stochastic transcription model predicts the presence of transcription traffic jams in the absence of GreA*_Spn_*. Data from simulations with and without GreA (mimicked by short- and long-stalling events) for different expression levels (in the simulations, we use the transcription rate as a measure of gene expression. This is proportional to the mRNA concentration measured in the experiments of Figure 3) (**A**) and different gene lengths (**B**). (**C**) Simulated elongation experiments: Amount of RNA synthesized as a function of time. The time that elapses between synthesis of an early probe (solid lines) and a late probe (dashed lines) reflects the elongation speed. Lack of GreA*_Spn_* results in a decrease of the number of elongation complexes that reach the late probe (compare to Figure 5C). (**D**) Graphical representation of simulation time courses (kymographs): Each green dot indicates an active elongation complex, red dots indicate stalled complexes. Traffic jams of active elongation complexes form transiently behind stalled complexes.

The above results suggest that GreA*_Spn_* augments transcription by restarting stalled elongation complexes. However, this does not intuitively explain why highly expressed genes are particularly sensitive to the lack of GreA*_Spn_* while longer genes are not (Figure 3). To strengthen this conclusion and to gain more insights in the molecular mechanism involved, we developed a stochastic model of transcription in the presence and absence of GreA*_Spn_*. We built a model upon a framework previously established to model the effects of pausing, termination and antitermination on rRNA transcription in *E. coli* (32). The model takes into account that GreA*_Spn_* does not affect initiation and promoter escape. We used the model to test three scenarios, where stimulation of transcript elongation by GreA*_Spn_* is due to either an increase of the stepping rate (the elongation rate without pauses), a reduction of the duration of the pauses or of the frequency of pauses (Figure 6, Supplementary Figure S3). Simulations of all three scenarios were consistent with the experimental observations that: highly expressed genes are more sensitive to the lack of GreA*_Spn_* (Figure 6A, Supplementary Figure S3A and D); and that absence of GreA*_Spn_* does not significantly impact the expression of longer genes (Figure 6B. Supplementary Figure S3B and E). However, only the simulations where GreA*_Spn_* was taken to affect pausing could recover the observed pattern of the elongation experiment and show that less RNAPs reach the end of the gene in the absence of GreA*_Spn_* as indicated by the lower slope in the accumulation of the 3′ probe’ (Figure 6C, Supplementary Figure S3C and F). The simulations cannot definitely distinguish whether GreA*_Spn_* reduced the duration or the frequency of pauses, although the agreement with the elongation experiments is slightly better for the pause-duration scenario. Based on the known mechanism of Gre-factors in *E. coli* (2,5,10), we consider a reduction of the pause duration as more likely. Moreover, simulations of the scenario of a reduced pause frequency requires very rare, but very long pauses in the wild type, for which we have no evidence. Thus, the simulations provide additional support for our interpretation of the data in Figures 4 and 5. Importantly, simulations explain the reduced transcription in the absence of GreA*_Spn_* by the formation of transcription ‘traffic jams.’ These traffic jams are formed by RNAPs that queue behind the paused elongation complex (Figure 6D). The model also predicts that averaged velocity of transcription elongation (i.e. including pauses and traffic jams) is the main rate-limiting factor for highly expressed genes (Supplementary Figure S4).

## DISCUSSION

Bacterial Gre-factors have been mostly studied in *E. coli* and it has become clear that, at least *in vitro*, Gre-factors stimulate RNAPs intrinsic proofreading activity and in that way speed up transcription and seem to be involved in nearly all steps of transcription: initiation, elongation and fidelity. The consequences of the lack of Gre-factor for the cell's physiology *in vivo*, however, have remained largely elusive. This can be partly explained because of the genetic redundancy present: *E. coli* contains two Gre-factors, GreA and GreB and several additional regulators, DksA, Rnk and TraR, which can also bind to the secondary channel of RNAP (15–17). Thus, interpretation of the physiological function of Gre has turned out to be difficult (18). Here, we studied the *in vivo* role of the Gre-factor of *S. pneumoniae*, an organism that only contains one Gre-factor, GreA*_Spn_* and no other homologs. Furthermore, the *S. pneumoniae* genome does not code for any other secondary channel binding homologs of Gre. The importance of a functional Gre-factor became immediately clear by analyzing cells of the *greA_Spn_* knockout mutant, which are severely perturbed in their growth (Figure 1). Importantly, we were not able to pick up fast growing suppressors by plate or liquid growth assays (data not shown), indicating that single mutations in RNAP or elsewhere in the genome cannot compensate for the loss of function of GreA*_Spn_*. Together with RNA-Seq and biochemical assays we obtained a clear picture of the *in vitro* and *in vivo* characteristics of GreA*_Spn_* and the ramifications for cells to live without GreA*_Spn_* (Figures 2–5). These data allowed us to formulate a stochastic mathematical model of transcription, which predicts that in the absence of GreA*_Spn_*, RNAP queues rapidly arise and that these traffic jams compromise gene expression (Figure 6). Interestingly, the model could also reproduce the observation that highly expressed genes were downregulated relatively more than lowly expressed genes (Figure 3). A likely explanation for this phenomenon is that highly expressed genes initiate transcription very frequently and thus the total transcription rate is rather limited by the elongation rate (Supplementary Figure S4), while lowly expressed genes fire transcription infrequently and their total transcription rates are thus limited by transcription initiation; which we show is not affected in the absence of GreA*_Spn_* (Figure 4).

RNAPs and ribosomes were proposed to cooperate to rescue backtracked RNAPs by ‘pushing’ them forward (26,45). This model however would predict that in the presence of excessive backtracking the highly expressed genes must be affected less by the absence of GreA than low-expressed genes, because the trailing RNAPs and ribosomes would ‘push’ paused RNAP from backtracking. Our data show the contrary pattern of transcription, suggesting that the cooperation of RNAPs and ribosomes to rescue backtracked complexes may not be efficient enough to suppress deletion of GreA*_Spn_*. Instead our results suggest that the paused RNAP rather causes queuing of the trailing RNAPs. The situation might be different for rRNAs that are not translated and are not reduced in the *greA_Spn_* mutant, whereas genes encoding ribosomal proteins are downregulated (Supplementary Table S4). The rate of transcription elongation on rRNAs is roughly 2-fold higher than that of protein coding genes (46,47). Fast transcription suggests smaller amount of pausing sites thus decreasing the chances of backtracking. In addition, extensive secondary structures forming in the nascent rRNAs would physically block extensive backtracking. These properties may alleviate the necessity of GreA*_Spn_*. Whether this hypothesis is correct requires further investigation.

Backtracked transcription elongation complexes, which can be resolved by Gre-factors, were proposed to cause DNA-damage by collisions with the DNA-replication machinery (48,49). The collisions may also be suppressed by point mutations in RNAP that were proposed to destabilize elongation complexes and/or reduce backtracking (48). The absence of slow-growth suppressors in the *greA_Spn_* knockout mutant however, suggests that *S. pneumoniae* RNAP cannot easily overcome backtracking possibly because it is poorly processive (e.g. compared to *E. coli* RNAP; (41)). Our data also suggest that queues of RNAPs, rather than single backtracked RNAP, may be a major problem for replication fork progression.

Transcription initiation is generally assumed to be rate limiting and the most regulated step of gene expression. Here, we present evidence that elongation of transcription can also determine the rate of gene expression and, thus, represent a major point for gene regulation in *S. pneumoniae* and possibly in other bacteria. Indeed, many transcription elongation factors such as λN and λQ (of bacteriophage λ), NusA and NusG (of *E. coli*) that affect transcription elongation have been shown to affect gene expression (50). Interestingly, it was recently shown that the transcription rate of eukaryotic RNAPII (Pol II) varies on genes demonstrating that elongation is also a regulated rate-limiting step during transcription in higher organisms (51).

**Figure 7.**
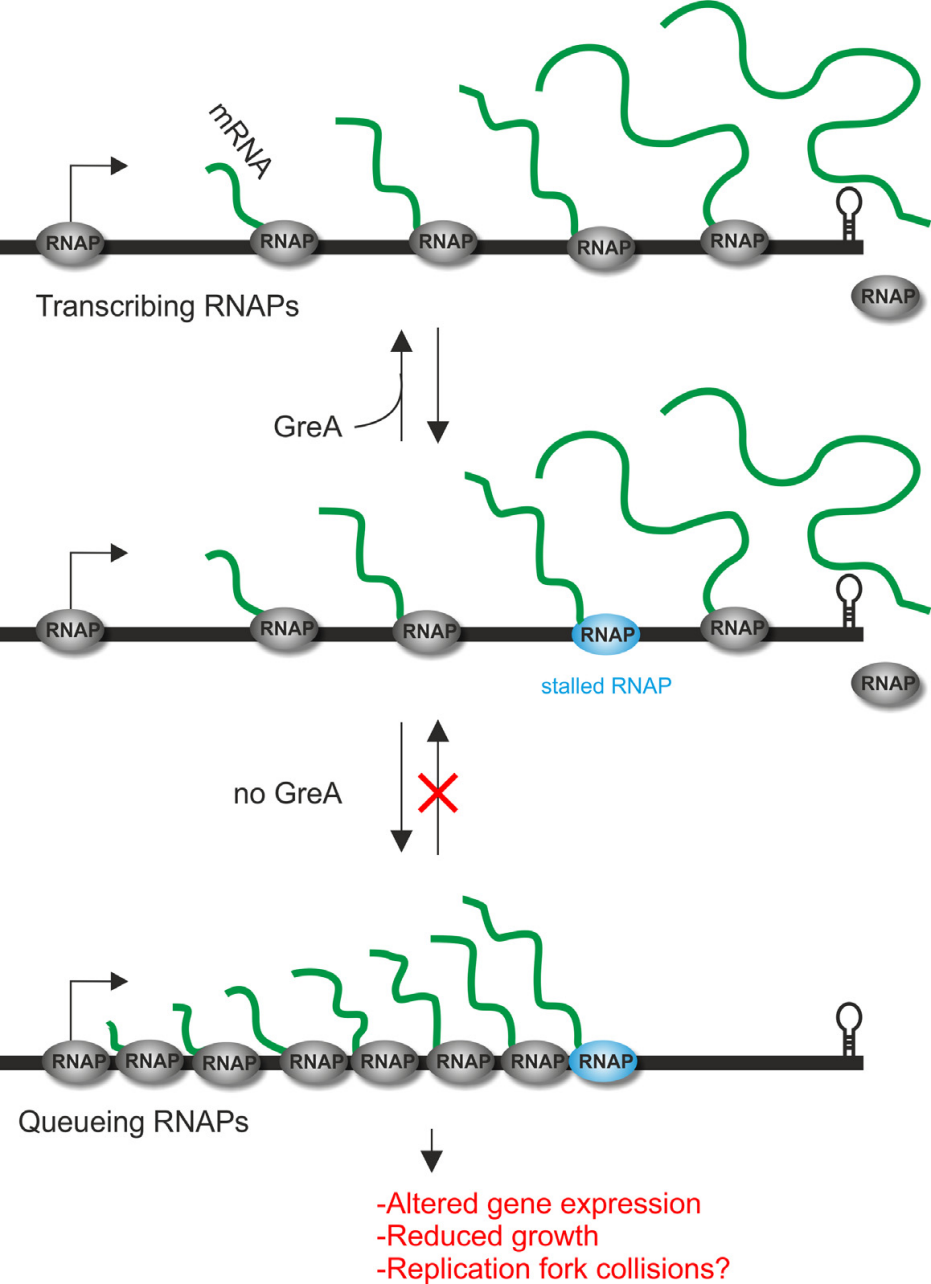
Model for the *in vivo* function of GreA*_Spn_*. RNAP stalls frequently, for instance due to misincorporation events. In the absence of GreA (red ‘do not enter’ symbol), queues of RNAP arise leading to altered gene expression and reduced growth. These stalled elongation complexes might also cause DNA-damage by collisions with the replication machinery (48,49).

Together, we now propose the following model for the major role of bacterial Gre-cleavage factors *in vivo* (Figure 7). After initiation of transcription and promoter escape, RNAP pauses on intrinsic signals (52) or as a result of misincorporation. These stalls, if not resolved, might lead to backtracking and cause queues of RNAP and, as a result, transcription ‘traffic jams’ arise (Figure 7). Importantly, these queues or traffic jams of RNAPs dramatically hamper gene expression and are detrimental for the cell. Future experiments, for instance chromatin immunoprecipitation assays using RNAP*_Spn_* antibodies and nascent transcript sequencing could be performed to test our model. Since our model predicts stochastic backtracking by RNAPs, single molecule and single-cell experiments might be more informative than bulk assays, and this is something we are currently pursuing. Indeed, nascent RNA-sequencing experiments showed no differences in RNAP*_Eco_* pausing in the absence of Gre indicating that backtracked pauses may occur randomly (52).

While *S. pneumoniae* provides a useful (minimal) model organism to study the bacterial transcription cycle *in vivo*, *S. pneumoniae* is also a serious human pathogen annually killing nearly 1 million children (53). Furthermore, over the last decades, *S. pneumoniae* resistance to existing antibiotics has spread and is now a serious problem (54). Our results reveal Gre-factor as a possible target for innovative drug design. Furthermore, our unique methodology, combining experimental methods on both the molecular (biochemical) level and the systems level and mathematical modeling, may serve as an example for studies on unrelated systems.

## SUPPLEMENTARY DATA

Supplementary Data are available at NAR Online.

SUPPLEMENTARY DATA
