# Supplementary material for: Control of transcription elongation by GreA determines rate of gene expression in Streptococcus pneumoniae
Source: Nucleic Acids Res. 2014 Sep 4;42(17):10987–99. doi: 10.1093/nar/gku790 (PMC4176173; doi:10.1093/nar/gku790)
Supplement: SUPPLEMENTARY DATA [file supp_42_17_10987__index.html]

Control of transcription elongation by GreA determines rate of gene expression in Streptococcus pneumoniae — Control of transcription elongation by GreA determines rate of gene expression in Streptococcus pneumoniae — SUPPLEMENTARY DATA 

# Control of transcription elongation by GreA determines rate of gene expression in *Streptococcus pneumoniae*

## SUPPLEMENTARY DATA

**Files in this Data Supplement:**

- SUPPLEMENTARY DATA
- SUPPLEMENTARY DATA
