# Supplementary material for: Control of transcription elongation by GreA determines rate of gene expression in Streptococcus pneumoniae
Source: Nucleic Acids Res. 2014 Sep 4;42(17):10987–99. doi: 10.1093/nar/gku790 (PMC4176173; doi:10.1093/nar/gku790)
Supplement: SUPPLEMENTARY DATA [file supp_gku790_nar-03219-x-2013-File009.pdf]

Supplemental information

to ‘Control of transcription elongation by GreA determines rate of gene expression in *Streptococcus pneumoniae*’ by Yuzenkova, Gamba *et al*.

Supplementary Experimental Procedures

Strains, plasmids and growth conditions

S. pneumoniae strains R6 and D39 were grown at 30°C or 37°C in C+Y medium (1) or in GM17 medium (2). Standard conditions, as described in (3) were used. Blood agar plates were made from Columbia agar containing 3% defibrinated sheep blood (Johnny Rottier, Kloosterzade, the Netherlands). For induction of P_{czcD} (here noted P_{Zn}), $ZnSO_4$ or $ZnCl_2$ was added to liquid medium and blood agar plates.

E. coli EC1000 was grown at 37°C in a shaking incubator in TY broth (Bacto-Tryptone (1%), Bacto-yeast extract (0.5%) and 1% NaCl). When appropriate, antibiotics were added in the following concentrations for *E. coli*: ampicillin (amp) at 100 µg/ml and spectinomycin (spec) at 50 µg/ml. For *S. pneumoniae*, the following concentrations were used: tetracycline (tet) at 1 µg/ml, trimethoprim (trmp) at 10 µg/ml, spectinomycin (spc) at 100 µg/ml, chloramphenicol (cm) at 4.5 mg/ml, erythromycin (ery) at 0,05 mg/ml, kanamycin (kan) at 250 mg/ml.

For *S. pneumoniae*, storage of mid-exponential phase cultures was done by growing cells in GM17 or C+Y at 37°C to an OD600nm of approximately 0.2

and 0.4, respectively. The cells were centrifuged for 2 min at 14000 rpm and the cell pellet was resuspended in a volume of fresh medium containing 14.5% glycerol (v/v) that would result in an OD600nm of exactly 0.2 and 0.4, respectively. The cells were then aliquoted and stored at -80°C.

For transformation of *S. pneumoniae*, cells were grown in C+Y at 37°C to an OD600nm of approximately 0.12, then 100ng/ml of synthetic CSP-1 was added and cells were incubated 10 minutes at 37°C. Transforming DNA was added to the activated cells and a 20-minute incubation at 30°C followed. Cells were then diluted 10 times in fresh C+Y and incubated for 1h at 37°C. Transformants were selected by plating in Columbia agar supplemented with blood and antibiotics when needed.

Recombinant DNA techniques and oligonucleotides

Common DNA procedures such as DNA isolation, restriction, ligation, gel electrophoresis and transformation of *E. coli* were performed as described (4). Chromosomal DNA of *S. pneumoniae* was isolated using the Promega Wizard Genomic DNA Purification Kit. Oligonucleotides used in this study are listed in Table S2 and were purchased from Biolegio (NL) or Metabion (DE). Enzymes were purchased from Roche (Mannheim, Germany), New England Biolabs (Ipswich, USA), Bioline (London, UK) and Fermentas (Burlington, Canada) and used as described by the manufacturer. For PCR amplification, Velocity polymerase (Bioline) or Pfu polymerase (Stratagene) were used.

Construction of plasmids and strains

Plasmids

To construct plasmid pJWV100, carrying a codon optimised variant of superfolder *gfp* (*gfp*(Sp), the synthetic construct from pUC57-gfp_sf was subcloned using the SphI/BlpI sites into similarly digested pPP2 (5) thereby replacing *lacZ* with *gfp(Sp)* flanked by terminators (6). To construct plasmid pJWV101, carrying the zinc inducible *czcD* promoter in front of *gfp*(Sp), plasmid pMP2 (7) was digested with BamHI/EcoRI and the P*czcD* (600bps) product was ligated into similarly digested pJWV100. The construction of plasmid pHK102 (P32-*gfp*) and derivatives carrying mutations in the -10 of the P32 promoter will be described elsewhere (Jorgensen, Karsens and Veening).

To construct plasmid pLA01, a PCR was performed using the primers p5-luc-F+BamHI and p5-luc-R+SpeI on the plasmid p5.00 (8). This PCR was purified and cut with BamHI and SpeI and cloned into pJWV100 cut BamHI/SpeI, thereby replacing the *gfp_sf* gene with the *luc* gene. To construct plasmids pLA13, the promoter of the late competence gene *ssbB* (P_{ssbB}) was amplified by PCR from D39 chromosome using the primers PssbB-F+NotI and PssbB-R+BamHI. This PCR was purified and cut with NotI and BamHI and cloned into pLA01 cut NotI/BamHI, thereby placing the *luc* gene under the control of the P_{ssbB} (pLA13) as described (9).

To construct plasmid pLA18 (9) allowing expression of both *luc* and a gene encoding a superfolder variant of GFP (*gfp*(Bs)) under the control of P_{ssbB},

gfp(Bs) was subcloned from pUC57-*gfp_sf_DSM* (6) using the XbaI/BlpI sites into similarly digested pLA13.

To construct plasmid pPGs4, carrying *greA* under the control of the Zinc inducible *czcD* promoter (P_{Zn}), *greA* gene was amplified with oligonucleotides sPG49 and sPG50, digested with *Bam*HI and *Spe*I and ligated to an equally cut pJWV101.

Plasmid pPGs6 carries a constitutively expressed *lacZ* reporter gene in which a STOP codon (TAA) has been introduced in place of codon 15. The P_{32}-$lacZ_{G15stop}$ cassette was amplified from plasmid pPGs3 (lab collection) with the oligonucleotide pair sPG57-sPG58, digested with *Bgl*II and *Spe*I and ligated to an equally cut pNZ8902 (10).

Plasmid pPGs9, carrying a catalytically inactive $greA_{Spn\text{-}D43A/E46A}$ mutant allele under control of P_{Zn}, was obtained by site-directed mutagenesis using plasmid pPGs4 as a template and by introducing the desired mutations with oligonucleotides sPG106 and sPG107.

Strains

S. pneumoniae strain PGs6 was constructed by replacing the *greA* gene with a chloramphenicol resistance cassette. Approximately 3,000 bp upstream and downstream *greA* coding sequence was amplified with the oligonucleotide pairs sPG13-sPG14, and sPG15-sPG16 respectively, using chromosomal DNA of strain D39 as a template. PCR products were digested with either *Asc*I or *Not*I and ligated to an equally cut chloramphenicol resistance gene, amplified from

plasmid pNZ8048 (11) with oligonucleotides sPG11 and sPG12. *S. pneumoniae* D39 was transformed with the ligation product, transformants were selected on GM17 agar plates supplemented with 1% defibrinated sheep blood and 2 µg/ml chloramphenicol. Gene replacement was verified by PCR.

S. pneumoniae strain PGs30 was constructed by replacing the *hexA* gene with a trimetoprim resistance cassette. Approximately 2,500 and 3,000 bp upstream and downstream *hexA* coding sequence was amplified with the oligonucleotide pairs sPG59-sPG61 and sPG60-sPG62 respectively, using chromosomal DNA of strain D39 as a template. PCR products were digested with either *Asc*I or *Not*I and ligated to an equally cut trimetoprim resistance gene, amplified from plasmid pKOT (12) with oligonucleotides 44-trmp-F+AscI and sPG65. *S. pneumoniae* D39 was transformed with the ligation product, transformants were selected on GM17 agar plates supplemented with 1% defibrinated sheep blood and 18 µg/ml trimethoprim. Gene replacement was verified by PCR.

S. pneumoniae strains PGs28 and PGs59 were constructed by transforming strains D39 or PGs30 with plasmids pPGs4 or pPGs9 respectively. Double crossover in *bgaA* locus was verified by PCR. Strains PGs44 and PGs45 were constructed by transforming strains D39 or PGs6 respectively with plasmid pLA13. Double crossover in *bgaA* locus was verified by PCR. Strains PGs48 and PGs67 were constructed by transforming strain PGs28 or PGs59 respectively with chromosomal DNA of strain PGs6. Strains PGs46 and PGs49 were constructed by transforming strain D39 or PGs6 respectively with chromosomal

DNA of a strain carrying an empty pPP2 plasmid integrated into the *bgaA* locus by a double crossover event, thereby inactivating the *bgaA* gene. Strains PGs50 and PGs52 were constructed by transforming strains PGs46 or PGs49 respectively with plasmid pPGs6. Strain PGs58 was constructed by transforming strain PGs30 with plasmid pPGs4. Strain PGs66 was constructed by transforming strain PGs58 with chromosomal DNA of strain PGs6. Strain PGs74 was constructed by transforming strain PGs66 with plasmid pPGs6.

Strains D39 and PGs6 were transformed with pLA13 to make strains PGs44 and PGs45 respectively and with pLA18 to make strains PGs57 and PGs65 respectively. For all these strains, selection of transformants was done on plate containing tetracycline and double crossover in *bgaA* locus was verified by PCR.

Strains SS1001-SS1004 were obtained by transformation *S. pneumoniae* with plasmids pHK102, pHK102B, pHK102C and pHK102G, respectively, and selecting transformants on plate containing tetracyclin. Double crossover in *bgaA* locus was verified by PCR.

Strains SS1005-SS1008 were obtained by transforming strains SS1001-1004 with chromosomal DNA of strain PGs6 and selecting transformants on plate containing chloramphenicol. The *greA* deletion was confirmed by PCR.

Fluorescence Microscopy

Cells were grown at 37°C in C+Y medium in half filled 5 ml capped tubes. Where relevant, Nile red (Invitrogen) was added to a final concentration of 8 ng/ml, and

DAPI was added to a final concentration of 0.2 μg/ml. 0.5 μl of the cell suspension was spotted on a microscope slide containing a slab of 1% PBS agarose. Images were taken with a Deltavision (Applied Precision) IX71Microscope (Olympus) using a CoolSNAP HQ2 camera (Princeton Instruments) with a 100X phase contrast objective. Emission/excitation filters were from Chroma. For DAPI, typical exposure times were between 1 and 2 seconds with 100% xenon light (300W). For Nile red, typical exposure times were 200ms with 32% of excitation light. Microscopy images modified for publication using ImageJ (http://rsb.info.nih.gov/ij/).

Growth curves, luminescence and fluorescence assays

C+Y medium was inoculated with mid-exponential phase frozen cultures. Cells were grown at 37°C in 96 wells plate (Polystyrol, white, flat and clear bottom, Corning) in a microtiterplate reader (Tecan Infinite F200 pro). Throughout the growth, absorbance (OD595nm), luminescence (expressed in RLU, relative luminescence unit) and fluorescence (arbitrary unit) were measured every 10 min. Expression of the *luc* gene results in the production of luciferase and thereby in the emission of light when the medium contains luciferin (13). For GFP fluorescence, excitation wavelength was 475nm and emission wavelength was 504nm. Other than shaking the plate for 10s prior to every measurement, no other measures were taken to maintain aeration since oxygen limitation is not an issue for *S. pneumoniae* which is a microaerophilic bacterium.

***In vitro* transcription assays**

For transcription from promoters, 0.1 pmole of PCR fragment carrying promoter was mixed with 0.3 pmole of $RNAP_{Spn}$ and 0.9 pmoles of σ^A with or without 0.1 pmole of $GreA_{Spn}$ in transcription buffer (TB; 33 mM Tris-Ac pH 7.9, 100 mM KGlu, 10 mM MgAc, 0.5 mM DTT, 0.1 mg/ml BSA). All reactions were performed at 37°C. After 10 minutes of open complex formation, reactions were started by addition of 0.5 mM ATP, GTP and CTP and 0.15 mM [α-^{32}P]-UTP (7,5 Ci/mmol), final, incubated for 10 min and stopped by addition of formamide-containing buffer. Products were resolved by denaturing (8M Urea) PAGE and revealed by Phosphorimaging (GEHealthcare).

Elongation complexes were assembled in TB lacking Mg^{2+} with 13 nt-long RNA radioactively labeled at the 5' end as described (14), except for complexes were immobilized on streptavidin agarose beads (Fluka) through biotin of the 5' end of DNA template strand (15). To form misincorporated complex (mEC14), 10 mM ATP and 20 mM $MgCl_2$ were added for 30 s. mEC14 was chased with 1 mM NTPs in the presence or absence of 10 nM $GreA_{Spn}$ for times indicated in the figure. Reactions were stopped and products analyzed as above.

Permanganate footprinting of open complexes was performed on promoters ^{32}P-kinased on either template or non-template strand, by addition of 5 mM $KMnO_4$ for 30 s, in the presence of 0.5 mM GTP (initiating nucleotide). Reactions were terminated by addition of β-mercaptoethanol to 330 mM, followed by phenol-extraction, ethanol-precipitation and 10% piperidine treatment.

RNA isolation, cDNA library construction and Illumina sequencing

Total RNA was isolated from mid-expontially growing cultures as described by (16). Sample preparation and sequencing were performed by Vertis (GER). In brief: the RNA samples were fragmented with ultrasound (7 pulses of 30 sec at 4°C) and then dephosphorylated with antarctic phosphatase followed by treatment with polynucleotide kinase (PNK). Afterwards, samples were poly(A)-tailed using poly(A) polymerase. Then a RNA adapter was ligated to

The 5´-phosphate of the RNA fragments. First-strand cDNA synthesis was performed using an oligo(dT)-adapter primer and the M-MLV reverse transcriptase. The resulting cDNAs were PCR amplified to about 30 ng/µl using a high fidelity DNA polymerase. PCR cycles were performed with barcode sequences, which are part of the 3' TruSeq sequencing adapter. The cDNAs were purified using the Agencourt AMPure XP kit (Beckman Coulter Genomics) and analyzed by capillary electrophoresis. For Illumina sequencing, the cDNA in the size range of 300 - 500 bp was eluted from preparative agarose gels. Aliquots of the size-fractionated cDNA were analyzed by capillary electrophoresis. The cDNAs are double stranded and have a size of about 300 – 500 bp. The primers used for PCR amplification were designed for TruSeq sequencing according to the instructions of Illumina. The following adapter sequences flank the cDNA inserts:

TrueSeq_Sense_primer 5´- AATGATACGGCGACCACCGAGATCTACACTCTT TCCCTACACGACGCTCTTCCGATCT-3’, TrueSeq_Antisense_primer Barcode 5’-CAAGCAGAAGACGGCATACGAGAT-NNNNNN-GTGACTGGAGTTCAGACG

TGTGCTCTTCCGATC(dT25)-3’. The combined length of the flanking sequences was 146 bases. For sequencing, the two cDNA samples were pooled in approximately equimolar amounts. The cDNA pool was then sequenced on an Illumina HiSeq 2000 system.

RNA-Seq data handling

Raw Illumina reads were quality assessed and trimmed using the Fastx toolkit version 0.0.13 from the Hannon Lab (http://hannonlab.cshl.edu/fastx_toolkit/index.html) and mapped using Bowtie version 0.12.7 (http://bowtie-bio.sourceforge.net/index.shtml,(17)). Bowtie settings were “-k 1 —best –strata”. To extract RPKM values for rRNAs (Table S4), Rockhopper was employed (18).

Calculating transcription mistakes using RNA-Seq data

Transcription errors were tallied using a custom Python script using the Bowtie output, by comparing the sequence of the mapped reads to the corresponding positions in the reference genome of *Streptococcus pneumoniae* D39 (NCBI annotation ID NC_008533.1, downloaded 20-2-2012; the python script is available on request. The errors were tallied with the assumption that the error-rate caused by the RNA Seq procedures itself would be equal for all samples using that same procedure, and would therefore not bias any comparison amongst them.

RT-qPCR

Strains D39 and Δ*greA* were grown in 50 ml C+Y acidic medium and samples for RNA isolation were taken between OD_{600} = 0.2-0.3 at their relative mid expositional growth phase and centrifuged for 2 min at 10000 g. Samples were immediately flash frozen in liquid nitrogen and stored at -80 °C. For RNA isolation, samples were thawed and dissolved in 400µl Diethyl-pyrocarbonate (DEPC) treated TE buffer (10mM TRIS-HCL 1mM EDTA, pH 7.5). Samples were added to a 2ml screw-cap tube on ice containing 500mg glass beads (75-150 µm), 150-175µl Macaloid, 50µl 10% SDS and 500µl phenol/chloroform mixture (1:1). Samples were placed in a Biospec Minibeadbeater-8 and beaten 2X 1 min with a 1 min interval on ice. Samples were centrifuged at 11000g for 10min at 4°C. The upper phase was added to 500µl chloroform and centrifuged for 5min at 11000g, 4°C. Further purification was done with the Roche High Pure RNA isolation kit using 1ml binding/lysis buffer. After purification, a second 1hr DNase incubation step was performed with DNase from the Roche kit, and the sample was purified a second time. For cDNA preparation, 2µl random nonamers (1.6µg/ml) were annealed to 10µg of total RNA in a total volume of 18µl for 5min at 70°C. Next, 12µl mastermix (6µl 5X First Strand buffer, 3µl 0.1M DTT, 25X dNTP mix (12.5mM each dNTP), 1.8µl Superscript III reverse transcriptase) was added and samples were incubated for 16hrs at 42°C. To degrade RNA, samples were treated with 3.5 µl 2.5M NaOH for 15min at 37°C. 15µl 2M HEPES free acid was added and samples were purified using a Nucleospin Gel and PCR clean up kit. RT-QPCR was done on a Biorad iQ5 PCR machine using Biorad iQ

supermix. Samples were diluted to 20ng/μl. Efficiency of the primers was experimentally determined to lie between 1.95 and 2.05. The relative gene expression was calculated using the Livak ($2^{-\Delta\Delta Ct}$) method (19). Primers used for RT-QPCR are listed in Table S6.

Stochastic transcription model

The model used to simulate transcription with and without GreA is based on the model of (20): Elongation complexes are described as stochastic steppers on a one-dimensional lattice representing the DNA and the lattice spacing corresponds to one nucleotide. Each elongation complex covers 50 sites. Elongation complexes move by stochastic single-site (single-nucleotide) steps, which occur with the elongation rate $\varepsilon \approx 50\ sec^{-1}$. If the target site (the site to which an elongation complex attempts to step) is occupied by another elongation complex, the stepping attempt is rejected (exclusion rule). Elongation complexes are initiated at the promoter with the rate α, provided that the promoter is free. This rate reflects the promoter strength and is varied to simulate genes with different expression levels. We refer to it as the initiation attempt rate, as the true initiation rate is smaller because the promoter is not always free. This rate is used to modulate the expression level in the simulations. Termination is assumed to be rapid once an elongation complex reaches the termination site at the end of the system (gene or operon). Stall events are allowed to occur at specific sites, which are selected randomly at the beginning of the simulation with an average distance of 30 between those sites. These sites mimic pause sites seen in the *in*

vitro transcription assays, but the presence of specific sites is not crucial for the results presented here. At these sites an elongation complex may enter the stalled state with rate *f*. Stalled elongation complexes are rescued and re-activated with rate $1/\tau$, where τ is the duration of the stall event. The key assumption of the model for studying the role of GreA is that the presence of GreA strongly reduces τ. As we do not have precise quantitative estimates for the pause parameters, simulations were performed with a wide parameter range. Results presented in the figures were obtained for $f=\varepsilon/100$, and $\tau=1000/\varepsilon$ (without GreA) or $\tau=1/2\varepsilon$ (with GreA), i.e. stalling durations of 20 sec and 0.01 sec. In addition, we test alternative scenarios where GreA is assumed to reduce the stepping rate ε or to increase the pause frequency *f* (Fig. S3). Qualitatively similar results are obtained for all situations where, for realistic initiation rates, transcription is limited by initiation in the presence of GreA, but becomes limited by elongation in its absence (Fig. S4). However, the scenario with a reduced stepping rate can be excluded as it is not consistent with the elongation experiment, as it shows a change of the slope of both the 3' and 5' probes as well as an increase in the time different between the first appearance of the probes (Fig. S3F). The results for a reduction of the pause frequency (Fig. S3 A-C) are similar to those for a reduction of the pause duration, although a small effect on the slope of the early probe, which is absent in the experimental data, is obtained in this case. Since Gre factors in *E coli* have been shown to stimulate transcript cleavage during backtracking pauses (14, 21), however, we consider shortening of the pause duration more likely.

The stochastic model was simulated with the kinetic Monte Carlo method described by Klumpp and Hwa (20) with Monte Carlo steps corresponding to a basic time unit $\Delta t \approx 0.01$ sec. Each Monte Carlo step consists of L moves, where L is the length of the gene (number of nucleotides). In each move a site is chosen randomly and updated depending on its occupation. We designate the position of an elongation complex by its rear end, i.e. an elongation complex at site x occludes sites x to x+49. If the chosen site x is occupied by an active (i.e. not stalled) elongation complex and site x+50 is free, the elongation complex moves to site x+1 (and now occludes sites x+1 to x+50) with probability $\varepsilon\Delta t$. If the site is one of the pause sites, the elongation complex may also enter the stalled state with probability $f\Delta t$. If the elongation complex at the chosen site is stalled, it may return to the active state with probability $(1/\tau)\Delta t$. If the chosen site x is free or occluded by an elongation complex located upstream of the site, no move is performed. Exceptions to these update rules are site 1 (the promoter) and the termination site L: If the termination site L is chosen, and an elongation complex occupies it, the elongation complex is terminated (removed). If the promoter is chosen and sites 1 to 50 are free, a new elongation complex is initiated at site 1. If an elongation complex occupies the promoter, it is updated in the same way as internal sites.

In all simulations we start with an empty lattice corresponding to a gene that is not transcribed. Simulations are run with two protocols: In the simulations for varying expression level (initiation attempt rate) and varying gene/operon length, we let the dynamics reach the steady state and then take time averages

over 4.5×10^6 Monte Carlo steps. In elongation experiment simulations, we record the number of elongation complexes passing through an early probe site and a late probe site to measure the time-dependence of the total transcription up to the current time in the transient dynamics immediately upon the start of transcription with an empty gene. The amount of RNA synthesized at each time point was obtained as an average over 1000 runs of the simulations.

The gene lengths (*L*) and initiation attempt rates (α) used to generate the data shown in the figures are: L=3000 in Fig. 6A, L=1800 in Fig. 6C and D; α=0.02, 0.2, and 2 sec^{-1} in Fig. 6B, and α=0.2 sec^{-1} in Fig. 6C and D.

Supplemental Figures

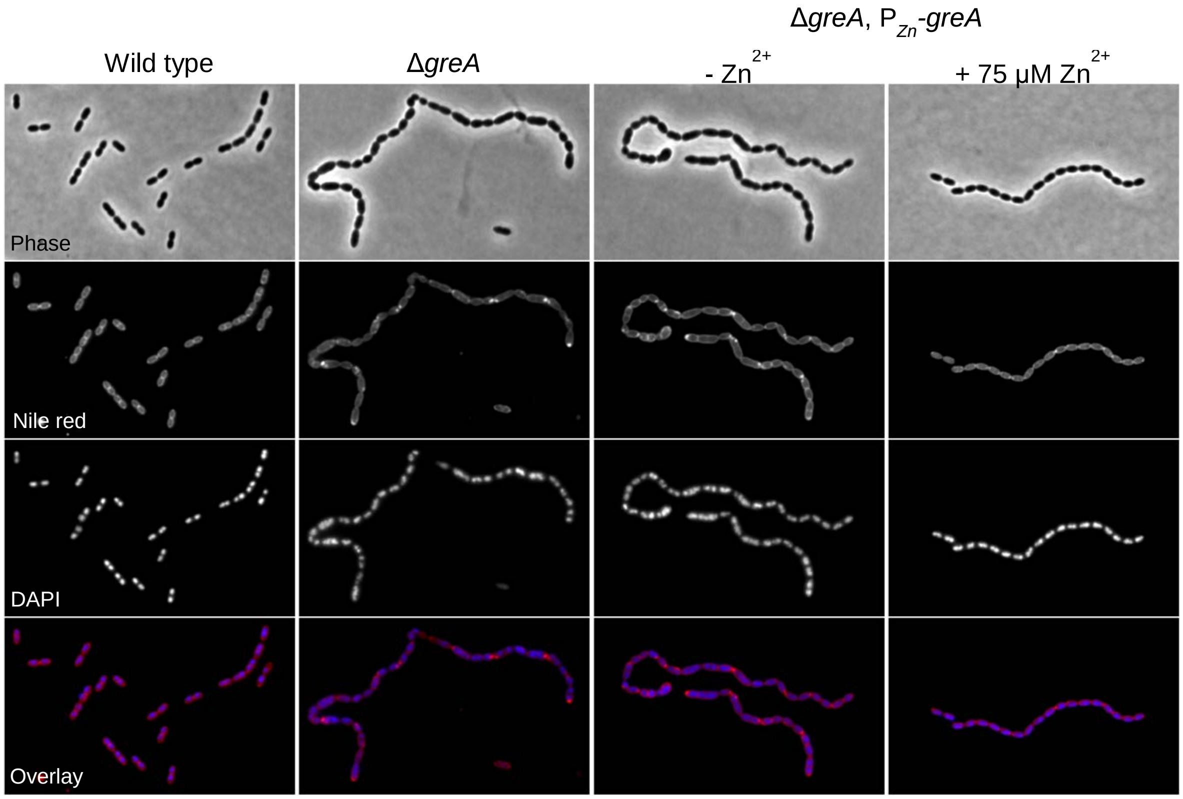

Fig. S1. GreA$_{Spn}$ is crucial for cell morphology. Microscopy analysis of wild type, the *greA* mutant and the complemented strain grown with or without 75 µM of Zn^{2+}. DNA was stained with DAPI and membranes with Nile red. Note that the addition of 75 µm Zn^{2+} induces cell chaining but it is clearly visible that at the individual cell level, complementation of *greA* restores normal cell morphology.

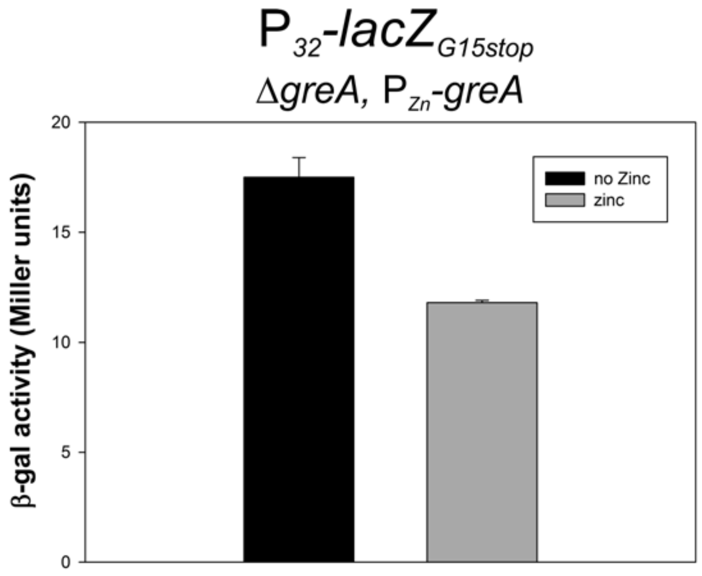

Fig. S2. Reduced *in vivo* DNA decoding fidelity in the absence of *greA*. Plasmid copy number was similar in both conditions (85.2 vs 81.2 plasmid copies per genome equivalent, with and without 75 μM Zn^{2+} respectively). Complementation of GreA increases DNA decoding fidelity *in vivo*.

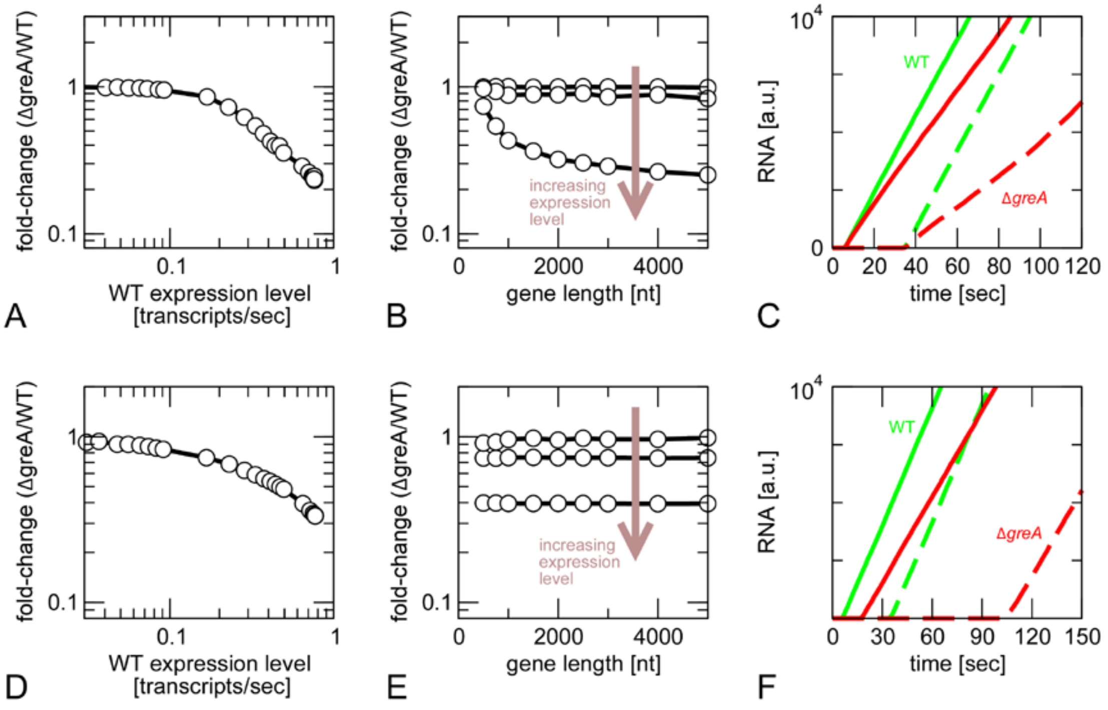

Fig. S3. Simulations assuming that GreA modulated the pause frequency *f* (A-C) or the stepping rate ε (D-F): Dependence on expression level (A,D) and gene length (B,E) and simulated elongation experiment with time dependence of the 3' and 5' probes. Simulations based on a GreA-modulated pause frequency (A-C) require long but very rare pauses in the WT ($f=\varepsilon \times 10^{-5}$ and $\tau=1000/\varepsilon$ for the WT), while for the strain without GreA the parameters are the same as in Fig. 6. The simulations in (D-F) use the parameters from Fig. 6 for WT and a 3-fold reduced ε for the strain without GreA.

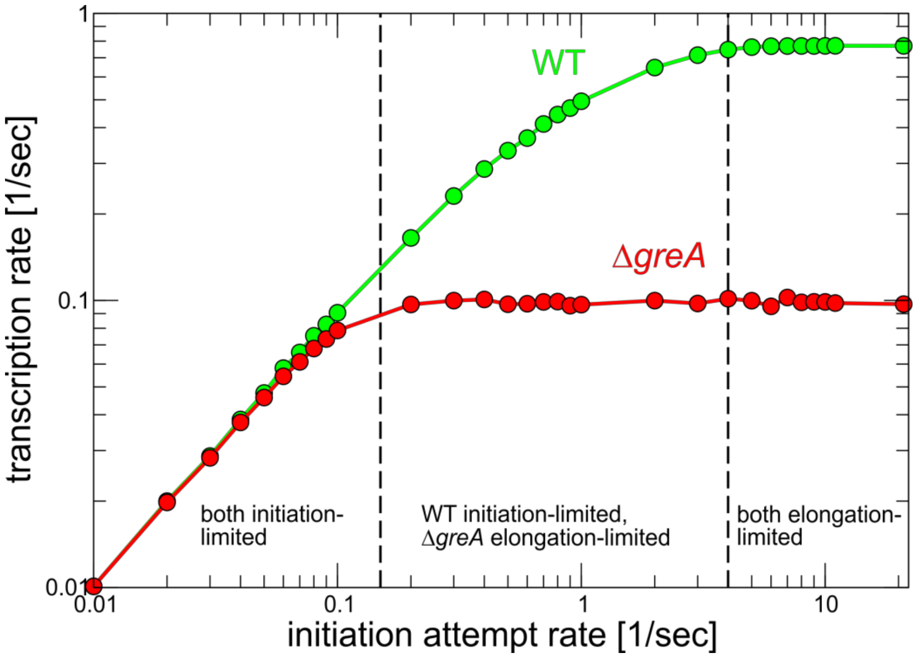

Fig. S4. Transcription rate and regimes of transcription in the stochastic transcription model. The transcription rate is plotted as a function of the initiation attempt rate (which reflects the promoter strength) for the wild type (green) and the mutant without GreA (red), which is implemented by slower re-activation of stalled elongation complexes. For low initiation attempt rates (weak promoters), the transcription rate is not affected by the duration of stalling events, as transcription is limited by initiation. For large initiation attempt rates, transcription is limited by (average) elongation (including pauses and traffic jams) and the gene is saturated with a lower saturated transcription rate in the GreA mutant than in wild-type. For intermediate initiation attempt rates, the mutant is saturated (elongation-limited), but the wild-type is still initiation-limited. This regime leads to the observed dependence of the transcription fold-change on expression level.

Supplemental Tables

Table S1: Functional classification of the number of genes significantly affected in Δ*greA*$_{Spn}$[a].

Functional categories	Total	Up	Down
[C] Energy production and conversion	14	4	10
[D] Cell cycle control, cell division, chromosome partitioning	3	2	1
[E] Amino acid transport and metabolism	31	16	15
[F] Nucleotide transport and metabolism	23	3	20
[G] Carbohydrate transport and metabolism	68	33	35
[H] Coenzyme transport and metabolism	12	9	3
[I] Lipid transport and metabolism	5	1	4
[J] Translation, ribosomal structure and biogenesis	16	4	12
[K] Transcription	23	10	13
[L] Replication, recombination and repair	20	13	7
[M] Cell wall/membrane/envelope biogenesis	14	6	8
[N] Cell motility	3	3	0
[O] Posttranslational modification, protein turnover, chaperones	13	8	5
[P] Inorganic ion transport and metabolism	24	19	5
[Q] Secondary metabolites biosynthesis, transport and catabolism	3	2	1
[R] General function prediction only	34	13	21
[S] Function unknown	26	8	18
[T] Signal transduction mechanisms	4	0	4
[U] Intracellular trafficking, secretion, and vesicular transport	5	3	2
[V] Defense mechanisms	17	11	6
Genes with no assigned COG categories	179	116	63
**Total number of genes**	**537**	**284**	**253**

[a] representing genes with at least 2 fold increase or 2 fold decrease in expression levels.

Table S2: Transcriptional changes in Δ*greA*$_{Spn}$ validated by RT-qPCR.

Gene name	Gene Function	Functional categories	Fold change wt/Δ*greA* RNA-Seq	qPCR
ccpA	catabolite control protein A	[K] Transcription	-2.59	-2
celA	competence protein CelA	-	38.97	4.5
SPD_0113	hypothetical protein	-	-189.3	-27.6
clpL	ATP-dependent Clp protease	[O] Protein turnover, chaperones	21.87	41.6
SPD_0267	xanthine/uracil permease family protein	[R] General function prediction	-10.16	-16.9
dnaA	Replication initiation protein	[L] Replication	1.34	4.6
purC	phosphoribosylaminoimidazole-succinocarboxamide synthase	[F] Nucleotide transport	-25.38	-16.2
SPD_0913	hypothetical protein	[S] Function unknown	13.4	33.1
htrA	serine protease	[O] Protein turnover, chaperones	6.42	14.4
greA	transcription elongation factor GreA	[K] Transcription	-5	-28215801

Table S3. Full list analyzed using bowtie downloadable as separate Excel file.

Table S4. Transcription of rRNA's is not significantly affected in the Δ*greA*$_{Spn}$ mutant. RPKM values for this table were determined using Rockhopper (18) and only ribosomal genes with an RPKM value of >500 in the wild type are listed.

Name	Product	RPKM wild type	RPKM Δ*greA*	Fold change
rrsA	16S ribosomal RNA	78055	73151	-1.07
rrlA	23S ribosomal RNA	60712	65516	1.08
rrsC	16S ribosomal RNA	51490	49331	-1.04
rrsB	16S ribosomal RNA	51442	49268	-1.04
rrsD	16S ribosomal RNA	51439	49392	-1.04
rrlC	23S ribosomal RNA	36161	40841	1.13
rrlB	23S ribosomal RNA	36150	40802	1.13
rrlD	23S ribosomal RNA	36119	40806	1.13
rpsU	30S ribosomal protein S21	2073	1286	-1.61
rplL	50S ribosomal protein L7/L12	1376	931	-1.48

rpsR	30S ribosomal protein S18	1217	688	-1.77
rpsA	30S ribosomal protein S1	1190	957	-1.24
rplQ	50S ribosomal protein L17	1029	661	-1.56
yfiA	Ribosomal subunit interface protein	998	219	-4.56
rpsO	30S ribosomal protein S15	973	856	-1.14
rplJ	50S ribosomal protein L10	892	633	-1.41
rpmG	50S ribosomal protein L33	869	666	-1.30
rpsT	30S ribosomal protein S20	839	312	-2.69
rpsM	30S ribosomal protein S13	830	570	-1.46
rpsQ	30S ribosomal protein S17	813	515	-1.58
rpmH	50S ribosomal protein L34	799	740	-1.08
rplX	50S ribosomal protein L24	793	539	-1.47
rpmD	50S ribosomal protein L30	773	416	-1.86
rplS	50S ribosomal protein L19	771	1006	1.30
rpmC	50S ribosomal protein L29	769	504	-1.53
rpmE2	50S ribosomal protein L31	714	589	-1.21
rpsH	30S ribosomal protein S8	687	372	-1.85
rplF	50S ribosomal protein L6	669	398	-1.68
rpsC	30S ribosomal protein S3	662	403	-1.64
rpsG	30S ribosomal protein S7	659	380	-1.73
rpsI	30S ribosomal protein S9	654	403	-1.62
rpsE	30S ribosomal protein S5	642	366	-1.75
rpsK	30S ribosomal protein S11	635	416	-1.53

rplE	50S ribosomal protein L5	626	359	-1.74
rpsS	30S ribosomal protein S19	620	463	-1.34
rplR	50S ribosomal protein L18	585	332	-1.76
rpmF	50S ribosomal protein L32	571	401	-1.42
rpmA	50S ribosomal protein L27	546	385	-1.42
rpsN	30S ribosomal protein S14	537	276	-1.95
rpsB	30S ribosomal protein S2	537	460	-1.17
rplP	50S ribosomal protein L16	529	359	-1.47
rplN	50S ribosomal protein L14	524	346	-1.51
rplM	50S ribosomal protein L13	505	278	-1.82

Table S5. Plasmids and bacterial strains used in this study

Strain/Plasmid	Relevant genotype*	Reference
E. coli		
EC1000	F$^-$, *araD139* (*ara ABC-leu*)*7679*, *galU*, *galK*, *lacX74*, *rspL*, *thi*, *repA* of pWV01 in *glgB*, Km^R	(22)
S. pneumoniae		
D39	Serotype 2 strain, *cps2*	(23)
PGs6	D39, Δ*greA*::*cat*	This study
PGs28	D39, Δ*bgaA*::(P_{Zn}-*greA*; *tet*)	This study
PGs30	D39, Δ*hexA*::*tmpr*	This study
PGs44	D39, Δ*bgaA*::(P_{ssbB}-*luc*)	This study
PGs45	D39, Δ*greA*::*cat,* Δ*bgaA*::(P_{ssbB}-*luc*)	This study
PGs46	D39, Δ*bgaA*::*tet*	This study
PGs48	D39, Δ*bgaA*::(P_{Zn}-*greA, tet*), *greA*::*cat*	This study
PGs49	D39, Δ*bgaA*::*tet*, *greA*::*cat*	This study
PGs50	D39, Δ*bgaA*::*tet*, pPGs6	This study
PGs52	D39, Δ*bgaA*::*tet*, *greA*::*cat*, pPGs6	This study
PGs57	D39, Δ*bgaA*::(P_{ssbB}-*luc-gfp*; *tet*)	This study
PGs58	D39, *hexA*::*tmpr*, *bgaA*:*Pcdz-greA*	This study
PGs59	D39, Δ*hexA*::*tmpr*, Δ*bgaA*::(P_{Zn}-*greA*$_{Spn\text{-}D43A/E46A}$; *tet*)	This study
PGs66	D39, *hexA*::*tmpr*, *bgaA*:*Pcdz-greA*, *greA*::*cat*	This study
PGs67	D39, Δ*hexA*::*tmpr*, Δ*bgaA*::(P_{Zn}-*greA*$_{Spn\text{-}D43A/E46A}$; *tet*), *greA*::*cat*	This study
PGs65	D39, Δ*greA*::*cat,* Δ*bgaA*::(P_{ssbB}-*luc-gfp*; *tet*)	This study
PGs74	D39 , Δ*greA*::*cat,* Δ*bgaA*::(P_{Zn}-*greA, tet*), Δ*hexA*::*tmpr*, pPGs6	This study
SS1001	D39 Δ*bgaA*::(P*32-(TATACT)-gfp*; tet)	This study
SS1002	D39 Δ*bgaA*::(P*32-(TACGCT)-gfp*; tet)	This study
SS1003	D39 Δ*bgaA*::(P*32-(TAAACT)-gfp*; tet)	This study
SS1004	D39 Δ*bgaA*::(P*32-(TATACT)-gfp*; tet)	This study
SS1005	D39 Δ*greA*::*cat* Δ*bgaA*::(P*32-(TATACT)-gfp*; tet)	This study
SS1006	D39 Δ*greA*::*cat* Δ*bgaA*::(P*32-(TACGCT)-gfp*; tet)	This study
SS1007	D39 Δ*greA*::*cat* Δ*bgaA*::(P*32-(TAAACT)-gfp*; tet)	This study
SS1008	D39 Δ*greA*::*cat* Δ*bgaA*::(P*32-(TATACT)-gfp*; tet)	This study
**Plasmids**		
pNZ8048	*E. coli-L. lactis* shuttle vector containing P_{nisA} promoter and start codon in NcoI	(11)

	site, *cat*	
pNZ8902	P_{spaS}, *ery*	(10)
pPP2	*bla*, *bgaA'*, *tet*, *'lacZ*, *'bgaA*	(5)
pKOT	*amp, trmp, pBluescript II KS*+	(12)
pJWV100	*amp*, *bgaA'*, *tet*, (no promoter) *gfp*, *'bgaA*	(6)
pJWV101	*amp*, *bgaA'*, *tet*, P_{czcD}-*gfp*, *'bgaA*	This study
pPGs4	*amp*, *bgaA'*, *tet*, P_{Zn}-*greA*, *'bgaA*	This study
pPGs6	*ery*, P_{32}-$lacZ_{G15stop}$	This study
pPGs9	*amp*, *bgaA'*, *tet*, P_{Zn}-$greA_{\mathrm{D43A/E46A}}$, *'bgaA*	This study
pJWV25	*amp*, *bgaA'*, *tet*, P_{Zn}-*gfp*, *'bgaA*	(24)
pHK102	pJW101 but P*32-(TATACT)-gfp*	This study
pHK102B	pJW101 but P*32-(TACGCT)-gfp*	This study
pHK102C	pJW101 but P*32-(TAAACT)-gfp*	This study
pHK102G	pJW101 but P*32-(TATACT)-gfp*	This study
pLA01	*amp*, *bgaA'*, *tet*, (no promoter) *luc*, *'bgaA*	This study
pLA13	*amp*, *bgaA'*, *tet*, P_{ssbB}-*luc*, *'bgaA*	(9)
pLA18	*amp*, *bgaA'*, *tet*, P_{ssbB}-*luc-gfp*, *'bgaA*	(9)

* *amp*, ampicillin resistance marker; *cat*, chloramphenicol resistance marker; *ery*, erythromycin resistance marker; *kan*, kanamycin resistant marker; *spec*, spectinomycin resistance marker; *tet*, tetracycline resistance marker; *trmp*, trimethoprim resistance marker.

Table S6 Oligonucleotides[a]

Primer	Sequence (5' to 3')	Restriction site
44-trmp-F+AscI	GCATGGCGCGCCGGATTTTTGTGAGCTTGGA	AscI
ccpA d	CTATAACTTTAGGAGGGCTTAGTTTCC	-
ccpA dn r	CATCTGCATTCATTCCTTTTCCTGTCC	-
ccpA fg r	GGTATTCATTAAGTCAACATCTCTTGTTTAAGAGC	-
CcpA-qt-Fr	CGTGATTCCAAATATTACCA	-
CcpA-qt-Rv	GGGTATTGACAACAGAAACA	-
ClpL-qt-Fr	CGCAGGTGTTGGTAAGA	-
ClpL-qt-Rv	GAGTACCAGCCTCAAGACC	-
CelA-qt-Fr	GTGCTGTCAAATCGCC	-
CelA-qt-Rv	CCAGAGCCTCATCACTAACT	-
Celab-qt-Fr	GCATTGGTGGCAAAAC	-
Celab-qt-Rv	CCAACAAAGCAAGATAGGA	-
czcD-F+FseI	GCATAGGCCGGCCTGTTAGTCATATGGACACTTAAGGC	FseI
czcD-R+EcoRI	GCGCGAATTCTTGTTATAATAGATTTATGAACACCTTG	EcoRI
DnaA-qt-Fr	GATGGAAATGTTTGGGC	-
DnaA-qt-Rv	GAATTTCATTTCCAATAGCG	-
gfp late start	CGGCATCAAAGCAAACTTCAAAATCCGTCATAACGTAGAGG	-
	ACGG	-
gfp late end t7	AAACGACGGCCAGTGAATTGTAATACGACTCACTATAGGC	-
	GCTTATAAAGCTCATCCATGCCGTGAGTGATACC	-
greA end	CTCCTTTTTCTCGAGTTAGGCTGTTTTTTC	XhoI
GreA-qt-Fr	CCTTCGACAAAGGCTTG	
GreA-qt-Rv	GTCCTCACCAATTTCTTGG	-
greA start	GAAGAAAGTTGAGACATATGGCAGAAAAAAC	NdeI
HtrA-qt-Fr	GGCAGGTTATCGGAATTAC	
HtrA-qt-Rv	CTGGATTCCCAAAGCTG	-
luc early start	AAACGACGGCCAGTGAATTGTAATACGACTCACTATAGGC	
	GGCAACTCCGATAAATAACGCGCCCAAC	
luc early end t7	GAGATCCGCCAAAAACATAAAGAAAGGCCCGGCGCCATTC	BamHI
p5-luc-F+BamHI	ATTAACCATGGGAGGATCCTAATTAGCTG	
p5-luc-R+SpeI	CGCGACTAGTGGATCTTACAATTTGGGCTTTCCGC	SpeI
PssbB-F-+NotI	CAAGGCGGCCGCCTCAGGATATTGCAGACACA	NotI
PssbB-R+BamHI	CGCAGGATCCGGTGTAGACGTTAAACGCCC	BamHI
purC d	CTGTGAGAAAGATTCTTCTTGCAG	-
purC dn r	CGCCTGGTCCTTGTAAGTTGAAATAATAAG	
purC full gene r	CCTTGAGGTTGTTATTTTAAACCCTGCAAC	
PurC-qt-Fr	GATGAGGGAATCGCCT	
PurC-qt-Rv	GGTCATCCGCAATCTGTA	-
SPD_0113qt-Fr	CCTACATTCCACAGACCATAG	-
rpoD sp xho r	CTTGATTCTCGAGGTCTTCAATAAAATCACGAAGCGG	XhoI
rpoD start nde	TACTGACCATATGGCAACAAAACAAAAAGAAGTAACAAC	NdeI
SPD_0264 d	GCAGGCAGAAAAGCAGATTCCAAAC	
SPD_0264 dn r	GCTCATTGCAAAGAAAGTTGTTAAACCAGCG	
SPD_0267 fg_r	GCCTGCACGATTCAACAATTCTTTCCAC	
SPD_0267-qt-Fr	CAATCTTTGGTGCAGCTC	
SPD_0113qt-Rv	GTTGAATCGTATCATGAATACC	-
SPD_0267-qt-Rv	CACCTGTACCGATCAAGG	-
SPD_0913-qt-Fr	GTGGCCAATGACTCTAGC	-
SPD_0913-qt-Rv	GGCTGCATTTCGGTACA	-
sPG11	ACGTGGCGCGCCAGGAGGCATATCAAATGAAC	AscI
sPG12	ACGTGCGGCCGCTTATAAAGCCAGTCATTAG	NotI
sPG13	ACGTGGCGCGCCCTCAACTTTCTTCTGATAAT	AscI
sPG14	TTATGGCTCGGCTCGTGAAG	

sPG15	AGATGCGGCCGCAAACAGAAAAAGGAGTGGGGAGGC	NotI
sPG16	ATTAGCGGTTGGCGAACTCC	
sPG49	AGCTGGATCCATTGAATCAGAAGAAAGTTGAGAAAAATG	BamHI
sPG50	ACTGACTAGTGCCTCCCCACTCCTTTTTCT	SpeI
sPG57	ATGCAGATCTAGGCCGGCCGATATGATAAG	BglII
sPG58	ACTGACTAGTACATAATGGATTTCCTTACGC	SpeI
sPG59	ACGAGGCGCGCCCTGTCTCTCCTTTTCAAAAA	AscI
sPG60	CAGTGCGGCCGCTGTCTTAGTAGAGTTAAAAC	NotI
sPG61	GCATATAAGCGGCTAGCGTC	
sPG62	CATCTCGTTGGTCGATTCTG	
sPG65	CAGTGCGGCCGCTTAACCGTTACGACGCGCATAGA	NotI
sPG106	GCCCGTTCATACGGTGCTCTTTCAGCAAACAGTGAGTACG AAGC	
sPG107	GCTTCGTACTCACTGTTTGCTGAAAGAGCACCGTATGAACG GGC	

[a] Relevant restriction sites are underlined.

References

1. Martin, B., Garcia, P., Castanie, M.P. and Claverys, J.P. (1995) The *recA* gene of *Streptococcus pneumoniae* is part of a competence-induced operon and controls lysogenic induction. *Molecular microbiology*, **15**, 367–379.

2. Terzaghi, B.E. and Sandine, W.E. (1975) Improved medium for lactic streptococci and their bacteriophages. *Appl.Microbiol*, **29**, 807–813.

3. Minnen, A., Attaiech, L., Thon, M., Gruber, S. and Veening, J.W. (2011) SMC is recruited to oriC by ParB and promotes chromosome segregation in *Streptococcus pneumoniae*. *Molecular microbiology*, **81**, 676–688.

4. Sambrook, J. (2001) Molecular cloning: a laboratory manual Cold Spring Harbor Laboratory Press, Cold Spring Harbor, NY.

5. Halfmann, A., Hakenbeck, R. and Bruckner, R. (2007) A new integrative reporter plasmid for *Streptococcus pneumoniae*. *FEMS Microbiol.Lett.*, **268**, 217–224.

6. Overkamp, W., Beilharz, K., Detert Oude Weme, R., Solopova, A., Karsens, H., Kovács, Á.T., Kok, J., Kuipers, O.P. and Veening, J.-W. (2013) Benchmarking various green fluorescent protein variants in *Bacillus subtilis*, *Streptococcus pneumoniae*, and *Lactococcus lactis* for live cell imaging. *Appl. Environ. Microbiol.*, **79**, 6481–6490.

7. Kloosterman, T.G., van der Kooi-Pol MM, Bijlsma, J.J. and Kuipers, O.P. (2007) The novel transcriptional regulator SczA mediates protection against Zn^{2+} stress by activation of the Zn^{2+}-resistance gene *czcD* in *Streptococcus pneumoniae*. *Mol.Microbiol.*, **65**, 1049–1063.

8. Chastanet, A., Prudhomme, M., Claverys, J.P. and Msadek, T. (2001) Regulation of *Streptococcus pneumoniae clp* genes and their role in competence development and stress survival. *J. Bacteriol.*, **183**, 7295–7307.

9. Slager, J., Kjos, M., Attaiech, L. and Veening, J.-W. (2014) Antibiotic-induced replication stress triggers bacterial competence by increasing gene dosage near the origin. *Cell*, **157**, 395–406.

10. Bongers, R.S., Veening, J.W., Van Wieringen, M., Kuipers, O.P. and Kleerebezem, M. (2005) Development and characterization of a subtilin-regulated expression system in *Bacillus subtilis*: strict control of gene expression by addition of subtilin. *Appl.Environ.Microbiol.*, **71**, 8818–8824.

11. Kuipers, O.P., de Ruyter, P.G.G.., Kleerebezem, M. and de Vos, W.M. (1998) Quorum sensing-controlled gene expression in lactic acid bacteria. *Journal of Biotechnology*, **64**, 15–21.

12. Hendriksen, W.T., Bootsma, H.J., Estevao, S., Hoogenboezem, T., de Jong, A., de Groot, R., Kuipers, O.P. and Hermans, P.W. (2008) CodY of *Streptococcus pneumoniae*: link between nutritional gene regulation and colonization. *J.Bacteriol.*, **190**, 590–601.

13. Prudhomme, M. and Claverys, J.-P. (2005) There will be a light: the use of luc transcriptional fusions in living pneumococcal cells. In *The Molecular Biology of Streptococci*. Hakenbeck,R., Chhatwal,G.S. (Eds) Horizon Scientific Press, pp. 519–524.

14. Zenkin, N., Yuzenkova, Y. and Severinov, K. (2006) Transcript-assisted transcriptional proofreading. *Science*, **313**, 518–520.

15. Roghanian, M., Yuzenkova, Y. and Zenkin, N. (2011) Controlled interplay between trigger loop and Gre factor in the RNA polymerase active centre. *Nucleic Acids Res.*, **39**, 4352–4359.

16. Van Hijum, S.A.F.T., de Jong, A., Baerends, R.J.S., Karsens, H.A., Kramer, N.E., Larsen, R., den Hengst, C.D., Albers, C.J., Kok, J. and Kuipers, O.P. (2005) A generally applicable validation scheme for the assessment of factors involved in reproducibility and quality of DNA-microarray data. *BMC Genomics*, **6**, 77.

17. Langmead, B., Schatz, M.C., Lin, J., Pop, M. and Salzberg, S.L. (2009) Searching for SNPs with cloud computing. *Genome Biol.*, **10**, R134.

18. McClure, R., Balasubramanian, D., Sun, Y., Bobrovskyy, M., Sumby, P., Genco, C.A., Vanderpool, C.K. and Tjaden, B. (2013) Computational analysis of bacterial RNA-Seq data. *Nucleic Acids Res.*, **41**, e140.

19. Livak, K.J. and Schmittgen, T.D. (2001) Analysis of relative gene expression data using real-time quantitative PCR and the 2(-Delta Delta C(T)) Method. *Methods*, **25**, 402–408.

20. Klumpp, S. and Hwa, T. (2008) Stochasticity and traffic jams in the transcription of ribosomal RNA: Intriguing role of termination and antitermination. *Proc. Natl. Acad. Sci. U.S.A.*, **105**, 18159–18164.

21. Borukhov, S., Sagitov, V. and Goldfarb, A. (1993) Transcript cleavage factors from *E. coli*. *Cell*, **72**, 459–466.

22. Leenhouts, K., Buist, G., Bolhuis, A., ten Berge, A., Kiel, J., Mierau, I., Dabrowska, M., Venema, G. and Kok, J. (1996) A general system for generating unlabelled gene replacements in bacterial chromosomes. *Mol Gen.Genet.*, **253**, 217–224.

23. Avery, A.T., MacLeod, C.M. and McCarty, M. (1944) Studies on the chemical nature of the substance inducing transformation of pneumococcal types. Induction of transformation by a desoxyribonucleic acid fraction isolated from Pneumococcus type III. *J.Exp.Med.*, **79**, 137–158.

24. Eberhardt, A., Wu, L.J., Errington, J., Vollmer, W. and Veening, J.W. (2009) Cellular localization of choline-utilization proteins in *Streptococcus pneumoniae* using novel fluorescent reporter systems. *Mol.Microbiol.*, **74**, 395–408.
